# Oxytocin substitution therapy in patients with AVP deficiency (central diabetes insipidus): study protocol of a double-blind, randomised placebo-controlled trial

**DOI:** 10.1136/bmjopen-2025-109940

**Published:** 2026-05-04

**Authors:** Cihan Atila, Svenja Leibnitz, Andi Nikaj, Matthias E Liechti, Dominique De Quervain, Mirjam Christ-Crain, the OxyTUTION Study Group

**Affiliations:** 1 Department of Endocrinology, Diabetes and Metabolism, University Hospital Basel, Basel, BS, Switzerland; 2 Department of Clinical Research, University Hospital Basel, University of Basel, Basel, BS, Switzerland; 3 Division of Clinical Pharmacology and Toxicology, Universitatsspital Basel Labormedizin, Basel, BS, Switzerland; 4 Division of Cognitive Neuroscience, University of Basel, Basel, BS, Switzerland

**Keywords:** Pituitary disorders, Clinical Trial, Clinical trials, General endocrinology

## Abstract

**Introduction:**

Arginine vasopressin (AVP) and oxytocin (OXT) are both hormones released from the posterior pituitary. While AVP primarily regulates water reabsorption in the kidneys, OXT plays a key role in socioemotional functioning. Due to the anatomical proximity, disruptions of the AVP system leading to AVP deficiency (AVP-D) may also affect the OXT system, possibly resulting in an additional OXT deficiency. This hypothesis was recently proven by using the 3,4-methylenedioxymethamphetamine stimulation tests and identifying OXT deficiency in patients with AVP-D, linked to increased anxiety and impaired emotion recognition. Despite these findings, OXT replacement therapy is not currently established as a treatment for AVP-D and long-term replacement therapy remains unexplored.

**Methods and analysis:**

This is a randomised, double-blind, placebo-controlled, parallel-group trial enrolling adults with AVP-D. Participants are randomised 1:1 to receive intranasal OXT (Syntocinon, 24 IU twice daily) or placebo for 28 days. The primary endpoint is a composite binary outcome defined as a clinically meaningful improvement in either trait anxiety (≥5-point reduction in State-Trait Anxiety Inventory-Trait Score) or emotion recognition (≥4-point increase in EmBody/EmFace task performance). Secondary outcomes include empathy, stress reactivity, neuroimaging markers of amygdala activity, additional psychological measures, metabolic parameters and safety outcomes, including hyponatraemia. Analyses will follow the intention-to-treat principle, with Fisher’s exact test used for the primary outcome and mixed-effects models for secondary endpoints.

**Ethics and dissemination:**

The study has been approved by the competent ethics committees and regulatory authorities in Switzerland and the European Union. The following institutions granted ethical approval: Ethikkommission Nordwest- und Zentralschweiz (EKNZ), project number EKNZ 2023–01010 and the Erasmus MC MERC, EU-CT number 2024–5 16 813-19-00. Results will be published in open-access, peer-reviewed journals and disseminated via scientific meetings, media communication and lay summaries provided to participants. De-identified individual participant data will be made available on reasonable request following publication.

**Trial registration number:**

NCT06036004.

**STRENGTHS AND LIMITATIONS OF THIS STUDY**

- The study uses a patient-centred design, with extensive patient and public involvement informing endpoint selection, study design, feasibility and outcome prioritisation, thereby enhancing clinical relevance and acceptability.
- A key limitation is the moderate sample size, inherent to rare-disease research, which may limit power for subgroup analyses and detection of small effect sizes, particularly for secondary and exploratory outcomes.
- The reliance on subjective psychological measures (eg, anxiety and empathy questionnaires) may introduce response variability; however, this is mitigated by the use of validated instruments and complementary objective computerised emotion-recognition tasks.
- Self-administration of the investigational medicinal product may lead to variability in adherence, which could affect treatment effects, although adherence will be monitored through diaries and medication accountability.

## Background

 Arginine vasopressin (AVP) and oxytocin (OXT) are closely related nine-amino acid neuropeptides.^1 2^ Both are synthesised in the hypothalamic supraoptic and paraventricular nuclei and released from the posterior pituitary.^1 2^ While AVP primarily regulates water balance, OXT plays a crucial role in regulating socioemotional behaviours, including attachment, fear reduction and emotion recognition.^1 3^

Disruption of the hypothalamic-pituitary axis due to tumours, inflammation or trauma can lead to AVP deficiency (AVP-D), formerly known as central diabetes insipidus, clinically characterised by hypotonic polyuria and polydipsia.^1 4^ Although desmopressin, an AVP receptor analogue, effectively controls these symptoms, patients frequently experience persistent psychological symptoms and impaired quality of life (QoL).^5^ Importantly, these findings on psychological symptoms are consistent across both, patients with isolated AVP-D and those with additional anterior pituitary dysfunction, therefore challenging the assumption that anterior pituitary hormone deficiencies alone contribute to these psychopathological changes.^5 6^ Given the structural proximity of AVP and OXT systems, disruptions causing AVP-D could also disrupt OXT production, potentially leading to a coexisting OXT deficiency. This disruption in the OXT system may—at least partially—explain the psychological symptoms observed in patients with AVP-D. This hypothesis is supported by 3,4-methylenedioxymethamphetamine (MDMA) stimulation tests, where healthy individuals exhibited an eightfold increase in OXT secretion, while patients with AVP-D showed no relevant OXT response.^7 8^ Importantly, in contrast to healthy controls, patients with AVP-D demonstrated reduced trust, impaired fear reduction and diminished recognition of positive emotions on MDMA stimulation, reinforcing the link between OXT deficiency and psychological impairments in this population. Despite these findings, OXT replacement therapy is not currently established as a treatment for AVP-D. Limited data from a single small study suggest that a single dose of intranasal OXT may improve emotion recognition, but its long-term effects remain unexplored. This randomised, placebo-controlled, double-blind trial aims to evaluate intranasal OXT therapy as a novel approach to improving psychological symptoms and socioemotional functioning in patients with AVP-D.

Intranasal OXT is the most commonly used formulation in behavioural research, as it enables partial direct brain delivery via olfactory and trigeminal pathways and is widely applied in human studies.^9^ Standard adult dosing in behavioural studies is 24 IU per administration, with reported doses ranging from 8 IU to 84 IU, and evidence suggesting a non-linear, inverse U-shaped dose–response with maximal central effects at 24 IU.^10 11^ In this study, Syntocinon (40 IU/mL) will be administered at a total daily dose of 48 IU, given as 24 IU twice daily (three puffs per nostril per dose). This regimen aligns with established dosing shown to increase cerebrospinal fluid OXT and modulate central emotional processing.

## Methods

### Study design

**Figure 1:**
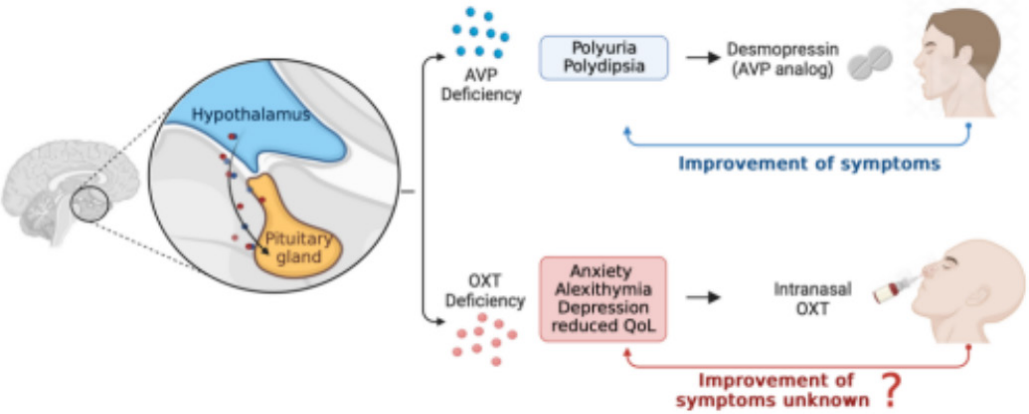
Main research question. AVP, arginine vasopressin; OXT, oxytocin; QoL, quality of life.

This is a randomised, double-blind, placebo-controlled, parallel-arm trial investigating the effects of intranasal OXT compared with placebo in patients with AVP-D (figure 1). The trial includes a 28-day treatment period, with patients randomised to receive either intranasal OXT (24 IU twice daily) or placebo. Participants will attend study visits on screening/day 0, day 1 and day 28 (±2). Optional substudies at day 14 will include fMRI (functional Magnetic Resonance Imaging) assessments and social-stress testing.

The trial is currently conducted at the University Hospital Basel, Switzerland, and Erasmus Medical Centre Rotterdam, the Netherlands, with additional study centres planned in Berlin, Germany and Lyon, France. Trial details are published on *Clinicaltrials.gov (NCT06036004) and Clinical Trials Information System (EUCT 2024–5 16 813-19-00*). The study protocol (V.5.0 (01.02.2025)) was approved by the relevant ethics committees and competent regulatory authorities in accordance with national and international regulations. In Switzerland, approval was obtained via the BASEC system (Business Administration System for Ethics Committees) from the competent Cantonal Ethics Committee (Ethikkommission Nordwest- und Zentralschweiz, No. 2023–01010) and Swiss Agency for Therapeutic Products (SwissMedic, No. 701718), and in the European Union, the study was authorised through the Clinical Trials Information System by the relevant national authorities (EUCT 2024–5 16 813-19-00). This study will be conducted in compliance with the protocol, the current version of the Declaration of Helsinki, ICH-GCP (International Council for Harmonisation – Good Clinical Practice), as well as all national legal and regulatory requirements.

### Eligibility criteria

See table 1 for the inclusion and exclusion criteria.

**Table 1:** Inclusion and exclusion criteria

Inclusion criteria	Exclusion criteria
Adult patients aged over 18 years with a confirmed diagnosis of AVP deficiency based on accepted criteria	Participation in a trial with investigational drugs within 30 days
Heightened anxiety levels (STAI-T subscale ≥39 score points) or alexithymia levels (TAS-20 total ≥52 score points)	Active substance use disorder within the last 6 months
Stable hormone replacement therapy for at least 3 months with desmopressin and, in case of additional anterior pituitary deficiencies, with the respective substitution therapies	Consumption of alcoholic beverages >15 drinks/week
	Current or previous psychotic disorder (eg, schizophrenia spectrum disorder)
	Pregnancy and breastfeeding within the last 8 weeks
	Unwilling to use an effective hormonal or mechanical form of contraception throughout the study period (female of childbearing potential only)
	Prolonged QTc-time >470 ms assessed with a 12-lead ECG

AVP, arginine vasopressin; QTc, corrected QT interval; STAI-T, State-Trait Anxiety Inventory-Trait; TAS-20, Toronto-Alexithymia Scale.

### Screening, recruitment and informed consent procedure

Participants will be recruited through endocrinologist referrals, electronic health records, online patient platforms, and previous or ongoing diagnostic studies for AVP-D. After initial contact and receiving brief information by telephone, potential participants will receive the patient information sheet and informed consent form by post or email. An example of the participant consent form is provided in the supplemental material. Interested patients may then contact the investigators to ask questions and arrange further evaluation.

**Figure 2:**
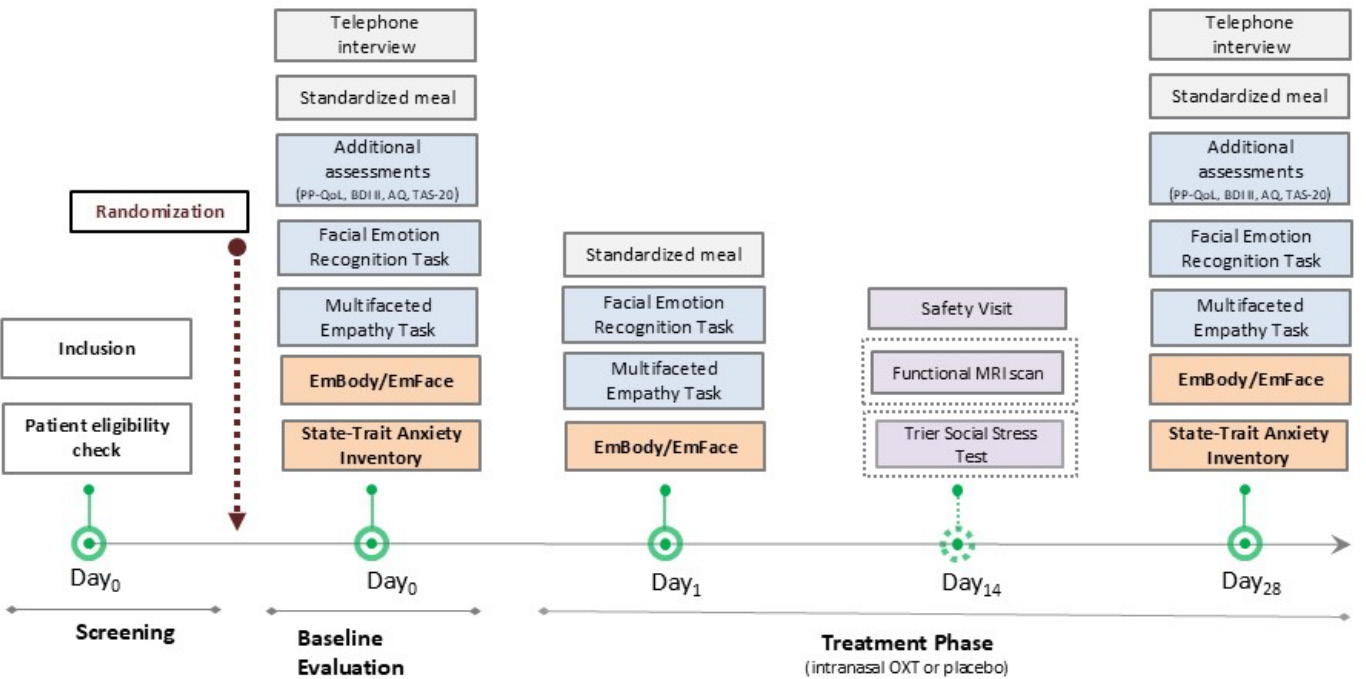
Study flow chart. AQ, Autism-Spectrum Quotient Test; BDI-II, Beck Depression Inventory; OXT, oxytocin; PP-QoL, Posterior Pituitary QoL Questionnaire; QoL, quality of life; TAS-20, Toronto-Alexithymia Scale.

At the screening visit, a study physician will explain the nature, purpose, procedures, duration, and potential risks and benefits of the study. Written informed consent will be obtained prior to any study-specific procedures. Screening assessments will include medical history, physical examination, standardised blood pressure measurement, body weight and height, and routine laboratory testing (blood chemistry, haematology and urine pregnancy test). A 12-lead ECG will be performed after at least 5 min of supine rest to exclude QT prolongation, with QT intervals corrected using Bazett’s and Fridericia’s formulas. Anxiety and alexithymia will be assessed using the State-Trait Anxiety Inventory-Trait (STAI-T) and Toronto-Alexithymia Scale (TAS-20) questionnaires, respectively. Participants meeting inclusion thresholds (≥39 in the STAI-T or ≥52 in the TAS-20)^12 13^ will subsequently undergo a standardised neuropsychiatric evaluation using the Mini-International Neuropsychiatric Interview and the Structured Clinical Interview for DSM-5 (Diagnostic and Statistical Manual of Mental Disorders, Fifth Edition) to screen for Axis I and II psychiatric disorders and a history of illicit substance dependence. All study physicians will receive dedicated training by a psychologist prior to study initiation to ensure standardised and consistent administration of these assessments. Final eligibility will be determined based on the combined clinical and psychiatric evaluation. Randomisation takes place immediately after completion of the screening visit, once a participant has been confirmed to fulfil all eligibility criteria and has provided written informed consent (figure 2).

### Randomisation and blinding

Participants will be randomised 1:1 to receive either intranasal OXT (Syntocinon) or placebo using the secure secuTrial system, employing block randomisation with a fixed block size of four and no stratification factors, to ensure balanced allocation over time while maintaining allocation concealment. Each randomisation number will correspond to a treatment package containing eight intranasal sprays (5 mL each) for a dosing regimen of 24 IU twice daily over 28 (±2) days.

The randomisation list is generated by the pharmacy and held by the Department of Clinical Research, Basel, and is concealed from participants, investigators, study nurses, study physicians, study psychologists and all outcome assessors. These individuals remain blinded throughout the trial.

Because the container systems of the OXT and placebo sprays differ slightly in appearance, unblinded study personnel, who are not involved in outcome assessments or clinical evaluations, are responsible for medication storage and dispensing. Participants are explicitly instructed not to show or discuss their study medication with blinded study personnel.

The placebo spray contains 0.9% sodium chloride with aromatic scenting to minimise sensory differences. With these procedures, blinding of participants and all personnel involved in clinical care, assessments and data analysis is maintained to ensure methodological rigour.

Emergency unblinding is foreseen in case of a positive urine pregnancy test in one of the main visits. In such a situation, the pregnancy will be confirmed by a blood test, further participation is not allowed, and the participant will be excluded. The online trial database secuTrial provides an emergency unblinding option which will be activated in agreement with the principal investigator in such case.

### Study intervention

#### Intervention group

This study will administer intranasal OXT spray, containing 40 IU of synthesised OXT per mL in a solution optimised for nasal absorption. Each spray delivers 4 IU of OXT per 0.1 mL, with each bottle containing 5 mL (200 IU total). Dr Hysek Pharmacy AG, Switzerland, will compound and supply the OXT spray under Good Manufacturing Practice standards. Participants will receive 48 IU of OXT daily, divided into two doses (24 IU in the morning and 24 IU in the afternoon), with three sprays per nostril administered per dose. Study personnel will instruct participants on administration techniques, emphasising a 45 s interval between sprays, and will provide dosing handouts and diaries to facilitate adherence tracking.

#### Control group

The placebo spray will be visually identical in size, volume and labelling but will differ in container system. It will contain 0.9% sodium chloride along with water for injection, disodium phosphate dodecahydrate, edetate disodium dihydrate and benzalkonium chloride. Each spray delivers 0.1 mL of placebo solution, with each bottle containing 5 mL. Packaging and labelling will ensure the sprays are indistinguishable to blinded study personnel.

#### Storage conditions

Investigational products are kept in a secure, limited-access storage area under recommended storage conditions at 2°C to 8°C in the refrigerator before opening. The bottle should be kept at room temperature (15°C to 25°C) after opening.

#### Blinding and dispensing

The dispensing of medication will be handled by unblinded study personnel. Participants will be instructed not to show their study medication to blinded personnel. Used and unused study medications will be returned to the unblinded personnel.

#### Modification, preventive measures and concomitant treatments

No dose modification is intended. No rescue medication for the trial participants is foreseen. In case of necessity of additional treatments during the intervention period, the participants will be instructed to document this in the drug-diary and inform the investigators accordingly. No concomitant care beyond the intervention is planned. Patients must be on stable hormone replacement therapy with desmopressin and, in case of additional anterior pituitary deficiencies, with the respective substitution therapies for at least 3 months before participation. If a change in replacement therapy is needed, each case will be discussed with the study team, and a possible change will preferably be planned before inclusion in the trial and there will be a wait of 3 months for stability. Concomitant psychotherapy (eg, medical therapy with psychotropics, behavioural therapy) is only allowed if this is established for at least 3 months before study inclusion and no discontinuation is planned during the intervention.

The first dose of the study medication will be given the day after successful inclusion. After that, study medication will be administered by the patient in the morning and afternoon daily at home. The empty carton containers with the eight sprays of the study medication must be returned to the investigator for compliance control at the follow-up visit on day 28 (±2). In addition, patients will be given a drug diary to document their use of IMP (Investigational Medicinal Product) and desmopressin.

### Data entry and quality control

Study data will be managed using the SecuTrial *Electronic Data Capture* system, hosted by the IT Department of the University Hospital Basel. The system automatically validates entered data for completeness and detects discrepancies. An audit trail records all entries and modifications, including reasons for changes, timestamps and user identification. Monitoring visits will be conducted regularly at the investigator’s site throughout the study. Source documents will be made available to monitors, and any queries will be addressed to ensure data accuracy and adherence to study protocols.

### Primary objective and outcome

The primary objective of this study is to assess the effects of intranasal OXT compared with placebo on anxiety levels and emotion recognition in patients with AVP-D. Clinically relevant improvement is defined as either:

- A decrease in the STAI-T Score by ≥5 points (range: 20–80)^12^
- An increase in EmBody/EmFace Task performance by ≥4 points (range: 0–42).^14^

The primary analysis will compare success rates—defined as achieving clinically meaningful improvement in anxiety or emotion recognition—between the OXT and placebo groups.

We hypothesise that the OXT group will exhibit a higher success rate than the placebo group. Additional details are provided in the online supplemental material.

*State-Trait Anxiety Inventory (STAI):* The STAI questionnaire evaluates anxiety through 40 items scored from 1 (‘almost never’) to 4 (‘almost always’), with total scores ranging from 20 to 80. It includes two subscales:

- STAI-S: Measures current anxiety (‘right now’), focusing on feelings such as nervousness or arousal.
- STAI-T: Assesses general anxiety tendencies or ‘anxiety proneness’.

A score above 39 indicates clinically significant anxiety. STAI-T will be evaluated on days 0 and 28, while STAI-S will be assessed on days 0, 14 (before and during TSST and fMRI) and 28.

*EmBody/EmFace tasks*: These tasks measure the recognition of emotions (angry, happy, neutral) in body and facial expressions through 42 standardised stimuli, each lasting 1.5 s. Participants select the perceived emotion in a three-option forced-choice format. Scores range from 0 to 42, with higher scores indicating improved recognition. Task design minimises biases (eg, ethnicity or clothing cues) and avoids consecutive repeats of the same actor, emotion or viewpoint. Each task takes approximately 5 min and will be conducted on days 0, 1 and 28.

### Secondary objectives

The three main secondary objectives are to determine whether OXT, as compared with placebo, has favourable effects on:

1. Emotion recognition and empathy
  - Correct recognition of facial expressions assessed on the Facial Emotional Recognition Task (FERT)
  - Levels in cognitive and emotional empathy assessed on the Multifaceted Empathy Task (MET)
2. Hormonal and subjective response to psychological stress (assessed on day 14 in a subset of patients, social-stress substudy)
  - Change in cortisol levels assessed on the Trier Social Stress Test (TSST)
  - Change in anxiety levels using the STAI (State-Anxiety Scale, STAI-S) on the TSST
3. Amygdala and subjective response in the functional MRI scan (assessed on day 14 in a subset of patients, fMRI substudy)
  - Amygdala response to the Emotional Face Matching Task (EFMT)
  - Change in acute anxiety levels using the STAI-S on the EFMT.

We hypothesise that intranasal OXT compared with placebo will, first, improve empathy and emotion recognition, second, reduce cortisol and acute anxiety levels in response to psychological stress, and third, reduce amygdala reactivity and acute anxiety levels in response to fearful faces.

Further objectives are to investigate whether the intervention as compared with placebo has effects on:

1. Other psychological measures (assessed on day 0 and reassessed on day 28):
  - Change in anxiety using the Hamilton Anxiety Rating Scale
  - Change in alexithymia using TAS-20
  - Change in autistic traits using the Autism-Spectrum Quotient Test (AQ)
  - Change in mood using the Beck Depression Inventory (BDI-II)
  - Change in QoL using the Posterior Pituitary QoL Questionnaire (PP-QoL) and the Patient-Reported Outcomes Measurement Information System (PROMIS)
  - Change in sexual desire (Sexual Desire Inventory-2, SDI-2) and in sexual behaviour (Sexual Behaviour Questionnaire, SBQ-G)
  - Change in eating behaviour using the Three-Factor Eating Questionnaire – Revised 18 item (TFEQ-T18)
2. Metabolic parameters (assessed on day 0 and re-assessed on day 28):
  - Change in weight
  - Change in HbA1c, glucose, insulin, C-peptide, lipid profile, IL-6, CRP (C-reactive protein), cortisol
  - Change in satiety and satiation by using numerical rating scales
3. Objective assessment of a third person (assessed on day 0 and reassessed on day 28):
  - Change in the objective assessment of a close third person regarding socioemotional characteristics using a predefined 20-item statement list with numerical analogue scales
4. Safety measure (assessed on day 0 and reassessed on day 14 and day 28):
  - Occurrence of hyponatraemia.

### Study procedure

See figure 2 and fiugre S1(online supplemental figure 1) for the study procedure.

*Baseline evaluation* (day 0, without IMP): Participants will complete the STAI-T, Hamilton Anxiety Inventory and TAS-20 questionnaires and perform computerised emotion recognition/empathy tasks (EmBody/EmFace, FERT, MET). Blood and urine samples will be collected for baseline analysis of hormones and metabolic markers. Participants will conduct self-reported questionnaires (STAI-S, BDI-II, AQ, PP-QoL, PROMIS), followed by an ad libitum meal to assess satiation. Close third-person assessments and questionnaires on sexual desire, behaviour (SDI-2, SBQ-G) and eating behaviour (TFEQ-18) may also be completed.

*Main visit 1* (day 1, with IMP): Participants will administer their first dose of intranasal OXT/placebo under supervision. Computerised tasks and additional questionnaires (STAI-S, subjective effects scales) will be completed at peak OXT concentration. Blood samples will be collected, and a second ad libitum meal will be provided to assess physiological and behavioural responses.

*Main visit 2* (day 28±2, with IMP): Primary outcomes (STAI-T and EmBody/EmFace tasks) will be completed. Participants will undergo final assessments, including blood sampling, IMP diary reviews, adverse event documentation and standardised questionnaires (eg, AQ, BDI-II, PP-QoL).

#### Optional substudies (day 14±2)

- Social stress (TSST): Participants will perform stress-inducing tasks under the supervision of a panel to assess cortisol and anxiety responses.
- fMRI: Participants will undergo a functional MRI using photographs from a validated set of facial stimuli that activate the amygdala and allowing isolation of amygdala reactivity to each emotional expression. Imaging will assess brain activity (resting state, EFMT) and structure. Blood samples and subjective questionnaires will complement the imaging data.

#### Concomitant desmopressin treatment

Hyponatraemia is a common complication of desmopressin therapy in AVP-D, affecting up to 27% of outpatients. To mitigate this risk, all participants will be instructed to use the ‘desmopressin escape method’, allowing breakthrough aquaresis before the next dose, which has been shown to significantly reduce hyponatraemia compared with rigid dosing. The OXT dose will remain stable throughout the study, while desmopressin dosing may be adjusted if needed; patients will be educated on symptoms of hyponatraemia, and daily desmopressin dose and fluid intake will be documented in a diary.

#### *Safety visit* (day 14±3)

Participants not involved in substudies will undergo sodium level monitoring either at the study site or by their healthcare provider. Plasma sodium will be assessed on day 14 and day 28 at the study site. If the patients are travelling from further distances, this control can be performed by their general practitioner or endocrinologist. Procedure if hyponatraemia occurs during the study:

- Mild hyponatraemia (plasma sodium 130–134 mmol/L): The patient will again be instructed in detail by our investigator on how to perform the ‘Desmopressin Escape’ method. We will follow-up on plasma sodium within the next 7 days.
- Moderate hyponatraemia (plasma sodium 125–130 mmol/L): Procedure see above. However, plasma sodium control is done earlier, after 2–3 days.
- Severe hyponatraemia (plasma sodium <125 mmol/L): The patients are monitored in the hospital until normalisation of the sodium value/symptomatic improvement after detailed assessment by the investigator.

### Sample size calculations and statistical analyses

The study will compare the success rates (SR) between the OXT and placebo groups. We hypothesise that the success rate is higher in the OXT group than in the placebo group. The null hypothesis is that the difference in the success rates between OXT and placebo (success rate difference (SRD)) is zero (H0: p_placebo=p_OXT, hence SRD=0).

For sample size estimation we used a simulation approach. We assumed a success rate of 10% under placebo and explored the sensitivity of the sample size to the expected success rate under OXT ranging from 20% to 50% (effect size θ=SRD 0.1–0.4). For each effect size θ and sample size N under consideration, we sampled 999 times from two binomial distributions with N/2 each and p placebo=0.1, and p OXT=0.1 + θ. The two proportions were then tested for a difference from zero (H0: SRD=0) using Fisher’s exact χ^2^-test at a significance level of α=0.05, using the chisq.test() function in R without continuity correction. The sample size is estimated to reject the null hypothesis with a statistical power of at least 85%.

For intranasal OXT, we expect a moderate effect, and a moderate effect size would also be regarded as clinically relevant. For binary outcomes, the effect size can be expressed as the SRD, that is, success rate under treatment minus success rate under control. The inverse of the SRD is the number needed to treat (NNT) which has a more clinically practical meaning. For example, a moderate SRD of 20% corresponds to an NNT of five patients. This means that five patients are needed to be treated in order to observe one patient with success. The sample size required depends on the true SRD θ. For an expected SR of 10% under placebo and 35% under OXT (SRD 0.25), a total of 112 patients (56 patients per group) should be recruited in order to end up with a total of 94 evaluable patients, considering a dropout rate of 15%.

#### Planned analyses

- Full analysis set (FAS): All randomised participants analysed as per intention-to-treat.
- Per-protocol set: Includes patients with psychological baseline assessments on day 0, primary outcome data on day 28 and ≥50% medication adherence.

The primary analysis will compare success rates between groups using Fisher’s exact test (α=0.05) on the FAS. Changes in STAI-T and EmBody/EmFace Task scores will be analysed using linear regression models, adjusting for baseline values. Results will include mean differences with 95% CIs. Secondary outcomes will be analysed using linear mixed-effects models for repeated measures, focusing on estimating OXT effects with 95% CIs. Adjustments for multiple testing will use the Bonferroni-Holm procedure. Descriptive statistics will summarise the outcomes for each treatment group.

#### Subgroup analyses

The primary outcome will be examined for effect modification by the following subgroups: sex (differences in the effects of intranasal OXT have been previously described), AVP-D (partial vs complete; hypothesis: stronger effect in complete AVP-D), aetiology group and concomitant pituitary dysfunction (isolated AVP-D vs combined anterior/posterior pituitary dysfunction; hypothesis: stronger effect in isolated AVP-D, since in combined pituitary dysfunctions the psychological changes might result from concomitant hormone deficiencies even under appropriate replacement therapies). Logistic regression models will be fitted with treatment group, subgroup and the interaction term. A strong interaction term would indicate an effect modification. However, the power to provide evidence for potential effect modification is limited to very strong effects. The subgroup analyses are, therefore, exploratory and hypothesis-generating. We will report the subgroup-specific observed success rates without inferential statistics.

#### Interim analyses

A data safety monitoring board (DSMB) will review safety data, including plasma sodium levels, after 60% of participants have completed treatment. The DSMB will consist of two independent neuroendocrinologists, one patient representative, and one data manager or statistician. The DSMB will provide recommendations regarding trial continuation, modification or termination in the event of safety concerns or serious adverse events. A prespecified safety signal is defined: if five or more additional cases of severe hyponatraemia occur in the OXT group compared with the placebo group, the DSMB will issue a recommendation for trial modification, which may include protocol amendments, enhanced safety monitoring, temporary interruption, depending on the clinical context. Access to interim safety data is restricted to the independent DSMB, while investigators and the study team remain blinded.

#### Handling of missing data and dropouts

According to the study design, only two main visits are planned, reducing the probability of missing data in the primary outcome. In case of missing data in the primary outcome we will apply best-case and worst-case imputations to examine the range of the potentially observed treatment effect.

### Data sharing statement

De-identified individual participant data that underlie the results of this study will be made available upon reasonable request to the corresponding author, following publication of the primary results. Data will be shared with qualified researchers for purposes of academic, non-commercial research, subject to approval of a methodologically sound proposal and completion of a data-sharing agreement. Requests will be evaluated in accordance with applicable ethical approvals, data protection regulations and institutional policies. Access will require approval and a signed data access agreement. The Department of Clinical Research, University of Basel, will act as an independent Data Access Committee and securely store the data at the time of publication. Requests for data reuse may be submitted via dkf.unibas.ch/contact.

### Ethics and dissemination

This study will be conducted in accordance with the principles of the Declaration of Helsinki and applicable Swiss regulatory requirements, including the Swiss Clinical Trials Ordinance (ClinO). The study protocol, informed consent forms and all relevant study documents have been approved by the competent ethics committee via the BASEC system prior to study initiation. Written informed consent will be obtained from all participants before any study-specific procedures are performed. Participants will be informed about the study objectives, procedures, potential risks and benefits, and their right to withdraw at any time without any consequences for their medical care. The risks associated with participation are considered minimal and are justified by the potential scientific and clinical benefits of improving the management of patients with AVP-D. All study procedures will be performed by qualified personnel, and participant safety will be continuously monitored. Participant confidentiality will be strictly maintained. Data will be coded and stored securely, with access limited to authorised study personnel. Handling of personal data will comply with applicable Swiss data protection regulations. Any substantial protocol amendments will be submitted to the ethics committee for approval prior to implementation.

The results of this study will be submitted for open-access publication in high-impact, peer-reviewed journals relevant to clinicians managing patients with AVP-D. For the primary trial results, a press release will be issued and distributed to Swiss and international media to ensure broad dissemination. In accordance with Swiss Clinical Trials Ordinance (ClinO) Art. 65 a, the sponsor will publish a summary of trial results in a public registry within 1 year of study completion or premature termination. A lay summary will also be entered into BASEC within the same timeframe and provided directly to study participants, in the national languages relevant to recruitment.

### Patient and public involvement

Patients and patient representatives were actively involved throughout the design and planning of the OxyTUTION Trial. Prior to protocol submission, patient advocates and representatives from international patient organisations collaborated with endocrinologists to develop and disseminate a large global web-based survey (>1000 participants) assessing psychological comorbidities in AVP-D; the results directly informed the study rationale and selection of patient-relevant outcomes.^5^ Patients and advocates further contributed to the selection and prioritisation of primary and secondary endpoints, testing the feasibility, relevance and burden of psychological tasks and questionnaires, and optimising study procedures, informed consent materials and investigational medicinal product instructions. Their feedback led to key design decisions, including adoption of a pragmatic parallel-group design over a cross-over approach. During the trial, patient representatives will contribute to safety oversight (including participation in safety monitoring discussions) and serve on the Trial Steering Committee to ensure continued incorporation of the patient perspective. After study completion, patients and advocates will be involved in interpreting and disseminating study results in accessible formats and in evaluating the impact of patient and public involvement to inform best practices for future research.

### Monitoring

Regular monitoring visits will take place at the investigator’s sites during the course of the study, as organised by the sponsor. The extent and nature of monitoring activities based on the objective and design of the study are defined in a study-specific monitoring plan. To this purpose, source data/documents are made accessible to monitors and questions are answered during monitoring. GCP-compliant monitoring will be performed by the Department of Clinical Research, University Hospital Basel.

## Discussion

This study protocol outlines a novel therapeutic option in patients with AVP-D, a condition marked not only by polyuria and polydipsia, but also by psychological comorbidities such as heightened anxiety and impaired socioemotional functioning. While desmopressin effectively manages the physical symptoms, it does not address the underlying psychological burden, leaving an unmet clinical need. This trial aims to bridge this gap by exploring OXT as a novel replacement therapy.

As the leading cause of disability and the third leading cause of overall disease burden, mental disorders are one of the most significant public health challenges (WHO). Therefore, investments in mental health improvement are essential for the sustainability of health and socioeconomic policies. High prevalences of psychological comorbidities in patients with anterior pituitary hormone dysfunctions are well recognised.^15 16^ However, research on AVP-D is generally limited because it is rare (approximately 15 000 patients in the EU) compared with other hormone dysfunctions, and because no commercial interests exist. Therefore, over the years, only limited research has been devoted to the role of OXT in patients with hypothalamic-pituitary dysfunction. Research primarily focused on patients with craniopharyngioma, a condition with a high risk of developing AVP-D.^17^ In these patients, a 10-year follow-up revealed personality changes (31%) and increased psychosocial comorbidities (47%),^18^ including anxiety, depression and social withdrawal.^17 19 20^ This was further confirmed by a systematic review showing neurobehavioral dysfunction in 57%, social impairment and difficulties holding relationships in 40%, heightened anxiety, depression and reduced social interaction.^21^ Multiple studies suggest that these symptoms may be linked to OXT deficiency.^522^,^24^ Additionally, impaired OXT release following physical stress in AVP-D/craniopharyngioma has been associated with autistic traits.^25 26^ These findings highlight the need for further investigation into the therapeutic role of OXT in patients with AVP-D.

Generally, treatment of anterior pituitary dysfunctions with hormone replacement therapy is strongly associated with symptom reduction or recovery. In AVP-D, on the other hand, only desmopressin is currently used for treatment, not addressing psychological changes associated with coexisting OXT deficiency. Studies have suggested that the function of OXT might be impaired in mental disorders associated with social deficits such as autism spectrum disorder, anxiety and depression, with conflicting results from treatment trials in these conditions.^27^,^37^ An important difference between these disorders and AVP-D is that no OXT deficiency per se has been proven; the evidence is largely based on observational studies with individual variations in peripheral OXT levels,^38^ in genes involved in OXT signalling^39^ or OXT receptor expression.^40^ In AVP-D, on the other hand, we have confirmed an OXT deficiency which is a strong rationale for treating these patients. The primary objective of our study focuses on determining whether OXT can achieve clinically meaningful improvements in anxiety levels and emotion recognition compared with placebo. This endpoint selection was based on previous interventional studies, demonstrating that intranasal OXT promotes prosocial effects such as ingroup favouritism,^41^ trust and attachment,^42^ empathy^43^ and emotion recognition.^44^ In addition, anxiolytic properties of OXT are well described, as it buffers responses to social stress,^45^ reduces amygdala response to emotional stimuli^46^ and reduces cortisol levels during conflict situations.^47^ The proposed dosing regimen of 48 IU/day, divided into two daily doses, is consistent with prior studies demonstrating the safety of OXT in other populations. Acute effects of intranasal OXT have been investigated in doses ranging from 6 IU to 84 IU. The most common dose for human behavioural research is 24 IU per dose.^48 49^ Comparison studies assessing central dose dependency in amygdala reactivity demonstrated the most pronounced effects 45 min after 24 IU of OXT (not following a linear dose-response and suggesting an inverse u-shaped dose effect with 24 IU as the most effective one).^50^ In line with this, various studies showed an increased OXT signal in the cerebrospinal fluid 75 min on intranasal OXT.^51^

*Patient and public involvement* played a central role in the design and planning of the trial. During the initial phase, a web-based survey, developed in collaboration with patient representatives, assessed psychological comorbidities. This survey, distributed through patient organisations, received responses from over 1000 patients worldwide, representing the largest data set on psychological well-being in AVP-D to date. Further input from patient representatives shaped the study’s primary and secondary endpoints, including the review of psychological tests, questionnaires and cognitive tasks. Notably, an initial cross-over study design was reconsidered based on patient concerns regarding time burden, potential dropout rates and carryover effects, leading to a more pragmatic approach. Patients actively participated in the study’s oversight through representation on the DSMB, where they contributed to the evaluation of safety aspects.

The lack of long-term data on OXT use in AVP-D highlights the need for safety monitoring of plasma sodium. Given the structural similarities to AVP, it is known that OXT affects urinary concentrations and has natriuretic effects due to crosstalk at V2 receptors (but with a lower affinity) in the kidneys.^52^,^57^ Thus, a synergic effect of OXT and AVP is possible and may reduce the needed desmopressin dose.^54^ Hyponatraemia is a common problem of desmopressin treatment, with a prevalence of up to 27% in outpatients.^58^ ^58^ We recently showed that performing the ‘desmopressin escape method’ led to a lower hyponatraemia prevalence than taking desmopressin on a rigid dose schedule.^5^ Therefore, all patients will be instructed to use this approach and will be educated on the symptoms and clinical signs of hyponatraemia.

One of the study’s strengths is its focus on exploring OXT’s effects in a specific, underserved population. By addressing both the psychological and physiological aspects of OXT deficiency, this trial could significantly advance the understanding and management of this novel condition, and the results could lead to a new treatment option for patients with AVP-D. Nonetheless, this study faces several challenges. First, even though randomised controlled trials are considered the gold standard for assessing causality, non-compliance with IMP self-administration may compromise outcomes. Second, reliance on subjective assessments of anxiety and emotion recognition introduces potential variability, though this is mitigated by using validated tools. Third, additional hormone deficiencies might have an impact on the results, though stable replacement for more than 3 months is a trial requirement, minimising this potential bias.

In summary, this study will be the first randomised controlled trial evaluating the effect of intranasal OXT on psychological comorbidities in patients with AVP-D. If the intervention with OXT leads to a significant improvement of anxiety and emotion recognition, the results of this trial could shape future management and potentially pave the way for a paradigm shift in the treatment of AVP-D. The study’s multicentre design provides a strong foundation for reliable and clinically meaningful evidence.

### Ethics statement

The study protocol received approval from the competent ethics committee of each participating centre, ensuring adherence to ethical guidelines and regulatory standards. The following institutions granted ethical approval: University Hospital Basel, Basel, Switzerland; Erasmus Medical Centre Rotterdam, Rotterdam, The Netherlands.

## Supplementary material

10.1136/bmjopen-2025-109940 online supplemental figure 1

10.1136/bmjopen-2025-109940 online supplemental file 1
