# Supplementary figures and images for: Oxytocin substitution therapy in patients with AVP deficiency (central diabetes insipidus): study protocol of a double-blind, randomised placebo-controlled trial
Source: BMJ Open. 2026 May 4;16(5):e109940. doi: 10.1136/bmjopen-2025-109940 (PMC13141166; doi:10.1136/bmjopen-2025-109940)

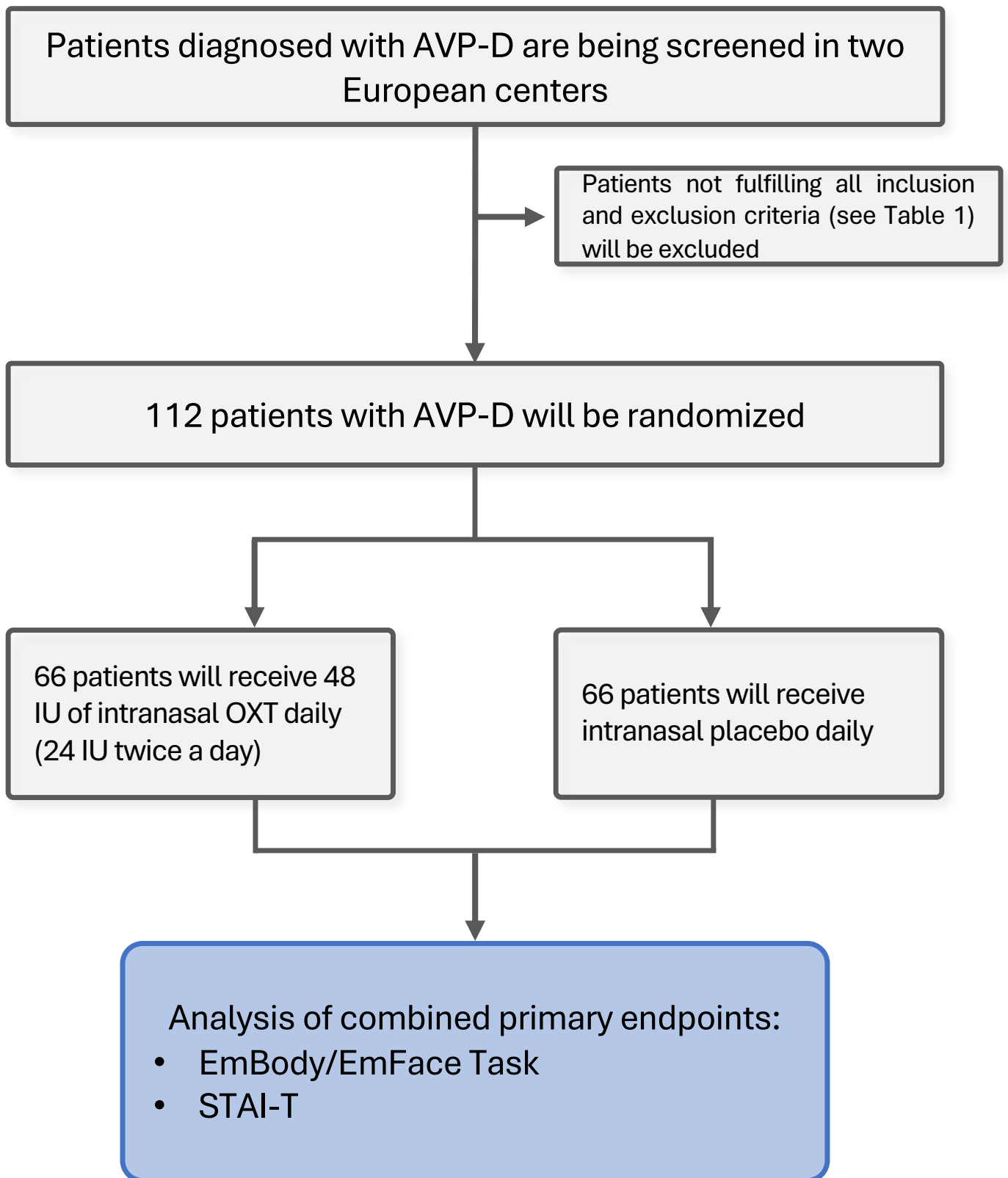

Supplement: online supplemental figure 1 [file bmjopen-16-5-s001.pdf]
