# Supplementary material for: Oxytocin substitution therapy in patients with AVP deficiency (central diabetes insipidus): study protocol of a double-blind, randomised placebo-controlled trial
Source: BMJ Open. 2026 May 4;16(5):e109940. doi: 10.1136/bmjopen-2025-109940 (PMC13141166; doi:10.1136/bmjopen-2025-109940)
Supplement: online supplemental file 1 [file bmjopen-16-5-s002.docx]

Inquiry Regarding Participation in Medical Research

**Study Title:** The **OxyTUTION** Study

**Layman`s Title**: Oxytocin nasal spray as a new treatment option for patients with AVP deficiency [Diabetes insipidus]

Dear Madam, Dear Sir,

hereby, we would like to inform you about this clinical study and inquire if you would like to participate. Before a new medication can be prescribed by doctors, the way this medication works must be researched.

Such research is called a **clinical study**. In this study, we aim to determine the effect of the study medication **oxytocin** on the well-being, psychological symptoms, emotion perception and empathy in patients with AVP deficiency [formerly known as diabetes insipidus].

You are suffering from AVP deficiency and are receiving treatment for it. Therefore, we are inquiring if you would like to participate in this study.

Your participation is voluntary. The following **patient information** is intended to assist you in making your decision. Any questions regarding the study participation can be **discussed with the investigator**, who is the doctor responsible for a study and who will be taking care of you during this study. If you wish to participate, please sign the **informed consent form** at the end. Your signature confirms that you have read and understood the patient information. If there is anything you do not understand, please ask the investigator.

The **patient information and informed consent form** consists of four parts:

**Part 1 Brief summary**

**Part 2 Detailed information about the study**

**Part 3 Data and insurance protection**

**Part 4 Informed consent form**

When you read **part 1**, you will get an overview of the study. In **part 2**, we explain the entire process and background of the study in detail. **Part 3** contains information about data and insurance protection. By signing at the end of the document in **part 4**, you confirm that you have understood everything and agree to participate.

The study is funded through an endocrinology fund by **Prof. Dr. Mirjam Christ-Crain**.

In the context of this study following person is responsible for you:

**Name**  Dr. med. Svenja Leibnitz **Emergency number** (available 24h):
**Address** Petersgraben 4, 4031 Basel Zentrale Universitätsspital:

**Phone**  + 41 61 32 8 44 96 +41 61 265 25 25

**E-mail** [svenja.leibnitz@usb.ch](mailto:svenja.leibnitz@usb.ch) ask for the on-call endocrinologist

Part 1: Brief summary

Vor allem ab Phase-3-Studien (IMP) oder konfirmatorischen MD-Studien.

# Why are we conducting this study?

Patients with AVP deficiency (central diabetes insipidus) suffer from a deficiency in the antidiuretic hormone **vasopressin**. Patients with such a vasopressin deficiency produce a large amount of urine and have to compensate for this fluid loss by consuming a high intake of liquids. Desmopressin, a medication similar to vasopressin, can be used to address this condition.

Despite treatment with Desmopressin, patients with AVP deficiency often report symptoms such as general anxiety, social withdrawal, low mood and have a reduced quality of life. A recent study has shown that these patients not only lack the hormone vasopressin but also the hormone **oxytocin**.

In the brain, oxytocin is produced at the same location as vasopressin and is released in the pituitary gland. This hormone is involved in important positive psychosocial aspects such as building trust, closeness, love and sexuality. It exhibits anxiety-reducing effects, positively influences sensations like relaxation, and enhances empathy—earning it the designation of the "*love and cuddle hormone*." The observed psychological changes in patients with AVP deficiency could potentially be explained by this additional deficiency in oxytocin.

In this study, we are investigating whether the administration of the **investigational drug Oxytocin** in patients with AVP deficiency leads to an improvement in psychological symptoms such as anxiety, emotion perception, social well-being, and ultimately enhances their quality of life. The goal is to determine if Oxytocin could be considered as a potential future treatment option.

# What do you need to do if you participate?

Your participation in this study will span approximately **28 (+/- 2) days**. We will invite you for **three study visits**. The first visit will last about 2-3 hours and the second and third visits about 2 hours each. Additionally, at the midpoint of the study (day 14 (+/-3)), a blood sample will be required – however, this can also be conducted locally, such as at your general practitioner’s office. You will discuss this option in more detail with your investigator.

Optionally, there is also an opportunity to participate in one or two additional study visit(s). Each of these would last approximately 2 (up to maximus of 3) hours.

If you decide to participate, you will be randomly assigned to one of **two groups**. You will either belong to the experimental group or the control group. Neither you nor the treating team will know which group you are in. In the experimental group, you will receive the study medication oxytocin as a nasal spray. In the control group, you will receive a nasal spray without oxytocin.

In **chapter 5**, you will learn more about the process and procedure of the study.

# What benefits and risks are associated with participating?

## Benefits

The direct benefit of the treatment to you is presently uncertain. Nonetheless, your participation holds the potential to contribute to advancements in treatment for future patients. The anticipated benefit is rooted in the possibility that the medication oxytocin may enhance mental well-being and overall quality of life for individuals with AVP deficiency. Previous case reports and studies involving ten patients have already highlighted positive effects on psyche and social behavior resulting from oxytocin. Additionally, studies involving patients with various conditions, such as depression or anxiety disorders, have shown favorable impacts of oxytocin.

## Riscs

The study medication oxytocin is currently approved in Switzerland primarily for obstetric use, specifically in inducing labor and supporting breastfeeding. However, its application in the treatment of patients with AVP deficiency has not undergone evaluation in a long-term study, and therefore, it is not yet sanctioned for use in this patient group in Switzerland.

You may experience side effects when taking the test substance oxytocin. Previous studies in patients with other diseases have shown no negative effects with the same dose of the test substance. The following risks and side effects have been described so far:

The study medication oxytocin is currently approved in Switzerland primarily for obstetric use, specifically in inducing labor and supporting breastfeeding. However, its application in the treatment of patients with AVP deficiency has not undergone evaluation in a long-term study, and therefore, it is not yet sanctioned for use in this patient group in Switzerland.

- Headaches
- Mild irritation of the nasal mucosa
- Inner restlessness
- Low blood salt levels (hyponatremia)

In **chapter 6**, you will find more information on risks and burdens.

Part 2: Detailed information about the study

# The scientific background of the study

## Background: Why are we conducting this study?

AVP deficiency (Diabetes insipidus) is a rare condition resulting from a deficiency of the hormone vasopressin. Vasopressin plays a crucial role in maintaining water balance in the body. A lack of vasopressin typically manifests as fluid loss through urine which is compensated by intense thirst and increased fluid intake. The condition is treated with the medication desmopressin.

For quite some time, there has been a suspicion that patients with a deficiency of vasopressin might also suffer from a deficiency of oxytocin. This suspicion is primarily rooted in the close proximity of the two hormone systems. A recently conducted study was able to confirm this and, for the first time, demonstrated an **oxytocin deficiency** in patients with AVP deficiency.

Oxytocin is a hormone produced in the brain and released into the bloodstream by the pituitary gland. Oxytocin is involved in important psychosocial aspects and has a positive impact on the mother-child relationship, closeness to a partner as well as sensations such as love, trust and relaxation. The hormone is frequently referred to as the *cuddle, love or loyalty hormone*.

A deficiency of oxytocin could therefore explain why patients with AVP deficiency often experience symptoms such as anxiety and depression as well as changes in social behavior with withdrawal, alterations in emotion recognition and empathy.

In this study, we are examining whether the administration of **oxytocin** to patients with AVP deficiency results in an amelioration of psychological symptoms, improvements in emotional and social well-being, and ultimately contributes to an enhanced quality of life. This investigation is being undertaken to explore the potential of oxytocin as a future treatment option.

## Study structure: What is our approach?

In our study, participants will be randomly assigned to **two groups**. This is important to obtain reliable results from the study. This process is called randomization. Each group will receive a different treatment. In our study, there are **two groups**:

- **Group 1** (experimental group) will receive the study medication as a nasal spray at a dosage of twice 24 IU (International Units) per day.
- **Group 2** (control group) will receive a placebo, which is a nasal spray without an active ingredient, also taken twice a day.

The study is a so-called double-blind study. "Double-blind" refers to a condition where neither those conducting the study nor the participants are aware of the group assignments. This means that participants are unaware of their group assignment, and investigators do not know to which group individual participants belong. In this way, all participants are kept "blind" to minimize potential influences on the study results. The concept of randomization and double-blinding allows for an objective evaluation of the true effectiveness and safety of the study medication oxytocin.

## Regulations for scientific research involving humans

We are conducting this study in strict compliance with Swiss laws, including the Swiss Federal Human Research Act and data protection regulations. Furthermore, we adhere to all internationally recognized guidelines. The study has undergone thorough review and approval by the responsible ethics committee and Swissmedic. It's important to note that our study is national in scope, exclusively involving participants in Switzerland..

A detailed description of the study can also be accessed on the Federal Office of Public Health's website at [www.kofam.ch](http://www.kofam.ch/), using the SNCTP registration number SNCTP000005641 or the BASEC number 2023-01010*.*

# Study procedure

## What do you need to do if you participate in the study?

Participation in the study is voluntary and lasts for **28 (+/-2) days**. You must adhere to the study schedule (🡪 Chapter 5.2) and follow all instructions provided by your investigator.

You must inform your study doctor if:

- your health condition changes, for example, if your health worsens or if you experience new symptoms. This also applies if you decide to discontinue the study prematurely (🡪 Chapter 5.3 and 5.4).
- during the study period, you are simultaneously under treatment from another doctor and have been prescribed new medications (including complemantary medicine).

Additionally, you must consider the following:

- You must effectively use contraception to prevent pregnancy during participation (🡪 Chapter 5.5).
- It is advisable to only consume moderate amounts of alcohol (<15 standard drinks per week) and completely abstain from drugs during the study.

## What occurs during the appointments?

Throughout your participation, you will attend **three study appointments—initially on two consecutive days** and at **the conclusion of the 28-day study period**. The first two appointments are expected to last around 2-3 hours each, while the third appointment will be approximately 3 hours. Additionally, a venous blood sample will be taken at the midpoint of the study (day 14, +/-3 days) to monitor blood salt levels. This blood test can also be conducted locally after consultation with your investigator, for instance, at your general practitioner's office.

Optionally, you have the choice to partake in one or two additional appointments at the midpoint on the **14th day**. Each of these supplementary appointments will last approximately 2 hours. The sequence of appointments is illustrated in the figure below.

**DAY 0**  During the preescreening examination, the following will be conducted:

- You will be informed about the study, questioned about any physical issues, your past and current medications and undergo a physical examination. The investigator will discuss the effects of the substance and your expectations with you.
- We will conduct a blood pressure measurement and record your height and weight.
- An electrocardiogram (ECG) will be recorded, which involves attaching electrodes to your chest, arms and legs to capture the electrical activity of the heart muscle on the skin’s surface.
- A pregnancy test via urine for women of childbearing age is mandatory.
- You will complete a questionnaire about your mental well-being and will also be interviewed by a psychologist.

If you meet all the criteria and are willing to participate in the study, you will be included/admitted to the study.

**DAY 0** After the prescreening examination, the following will be done:

- You will be randomly assigned to either the control group (nasal spray without active ingredient) or the experimental group (nasal spray with oxytocin).
- You will complete various questionnaires about your mental well-being.
- A computer test will be conducted to assess empathy and emotion recognition. In this test, various body and facial expressions with emotions such as *joy* and *fear* will be presented to you and you will need to identify them based on your perception. This computer test will take approximately 30 minutes.
- Following this computer test, you will be offered a standardized meal.
- Furthermore, we will inquire if a telephone interview can be conducted with a person close to you (for example, a partner, parent, or sibling). During this telephone interview, we will assess your personality traits, such as "closeness to others" or "empathetic behavior," from the perspective of the person close to you. This interview will be repeated at the end of the study to capture any changes.
- You will receive two optional questionnaires on the topics of sexuality and eating behaviour, which you can complete at your leisure at a later date and bring or send to us on the following day.

**DAY 1** On the following day following the prescreening examination, the following will be conducted:

- A venous catheter will be inserted for blood sampling.
- We will provide a demonstration on the correct application of the nasal spray and guide you on maintaining a diary throughout the study.
- We will explain the “Desmopressin Escape” method to better control your necessary Desmopressin dose during the study, minimizing the risk of overdosing. Overdosing could lead to low sodium levels (blood salt levels).
- You will complete various questionnaires about your mental well-being.
- You will be asked to self-administer the first dose of the nasal spray.
- Approximately 40 minutes later, a computer test will be conducted to assess empathy and emotion. This test involves the presentation of various body and facial expressions depicting emotions such as joy and fear, and you will be required to identify them based on your perception. The computer test will take approximately 30 minutes.
- Following the computer test, you will be offered a standardized meal.

**DAY 14** (Optional: MRI-test) You have the opportunity to participate in an additional study day with the following activities:

- You are expected to arrive approximately 1 hour before the examination to complete various questionnaires related to your mental well-being, each taking about 5 minutes. A venous catheter will be inserted for blood sample collection.
- Following this, you will be asked to administer your daily nasal spray dose under supervision.
- About 40 minutes later, an examination of brain activity will be conducted using magnetic resonance imaging (MRI). During this test, the activity of your emotional center in the brain will be assessed in response to various emotional stimuli. Tasks will be displayed on a screen, and simultaneous MRI scans of your brain will be taken.
- During the examination, you will be shown images of various facial expressions depicting emotions such as fear, anger, and joy. Your task will be to identify these emotions based on your perception.
- After the MRI examination, you will once again complete questionnaires about your mental well-being, and a second blood sample will be taken.
- This examination will last approximately 90 minutes.

**DAY 14** (Optional: stress test) You have the opportunity to participate in an additional study day with the following activities:

On this study day, after a brief preparation period in a serene room, you will transition to another room to deliver an impromptu speech on a specific topic to members of our study team. The objective is to analyze hormone release during this stress response. Throughout your presentation, you will also field questions. Following each brief presentation and question session, you will have the opportunity to return to the initial quiet room. There, you can complete questionnaires and provide saliva samples at various time points—initially at the beginning of the examination, then immediately before and during the task. These samples will be used to measure cortisol, the stress hormone.

- You are expected to arrive approximately 1 hour before the examination to complete diverse questionnaires about your mental well-being, with each questionnaire taking about 5 minutes. A venous catheter will be utilized for blood sample collection.
- Upon your arrival at our study center, you will self-administer your daily nasal spray dose under supervision.
- This additional examination aims to provide insights into the potential impact of oxytocin on situations involving stress. The hypothesis is that oxytocin may mitigate the stress reaction.

If you are interested in participating **in both optional study** **days,** the examinations will be conducted on separate days.

**DAY 28** On the last day of the study, we will repeat the procedure carried out on both day 0 and day 1.

Schedule: General and addtitional examinations

| **Study visits/Appointments** | **Prescreening examination &**  **Appointment 1** | | **2** | **Safety**  **visit** | **(optional)** | **(optional)** | **3** |
| --- | --- | --- | --- | --- | --- | --- | --- |
| **Date (days from study start)** | **0** | **0** | **1** | **14**  **(± 3)** | **14**  **(**± **2)** | **14**  **(**±**2)** | **28**  **(**±**2)** |
| **Duration (hours)** | **1** | **2** | **2** | **0** | **2** | **2** | **2** |
| Informed consent discussion / Consent for study participation | 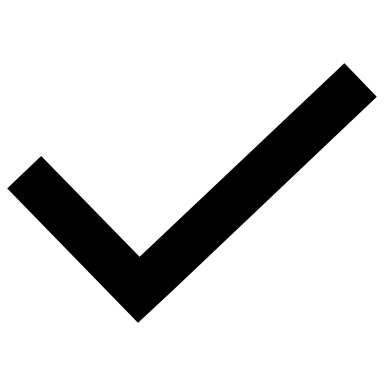 |  |  |  |  |  |  |
| Physical examination und interrogation | 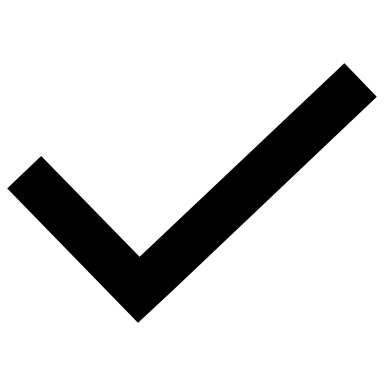 |  |  |  |  |  |  |
| Psychological interview | 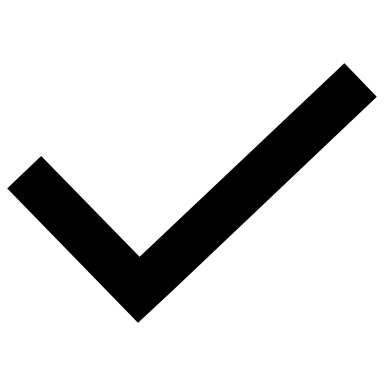 |  |  |  |  |  |  |
| Group assignment (oxytocin or placebo) |  | 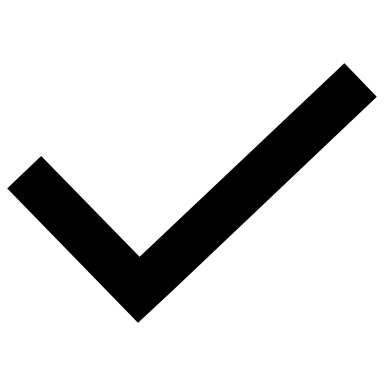 |  |  |  |  |  |
| Questionnaires | 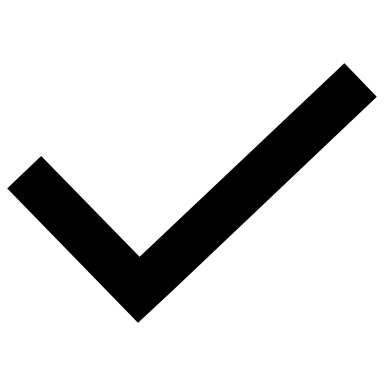 | 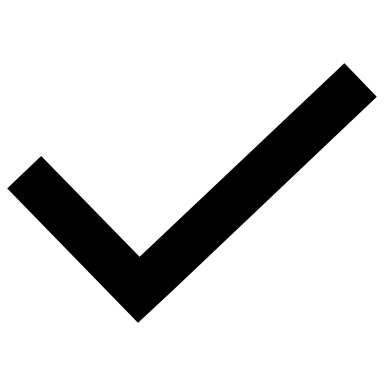 |  |  | 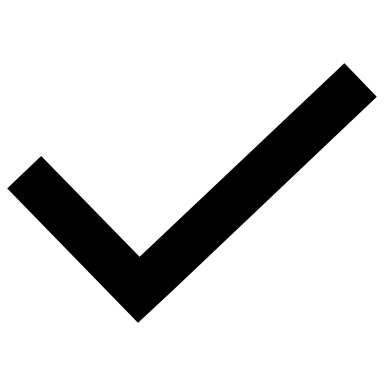 | 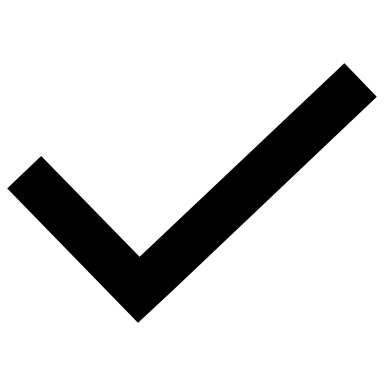 | 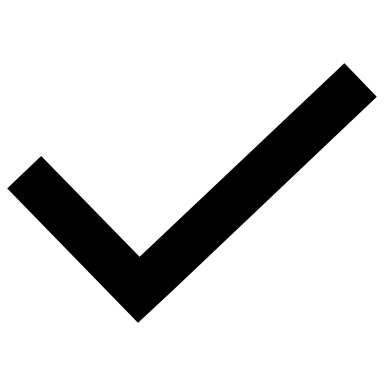 |
| Computer tests |  | 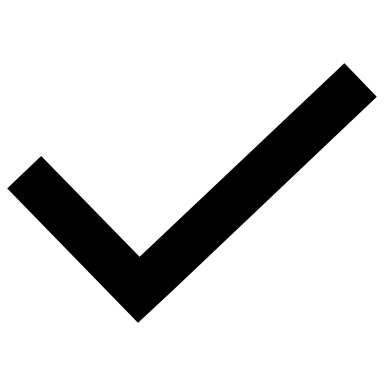 | 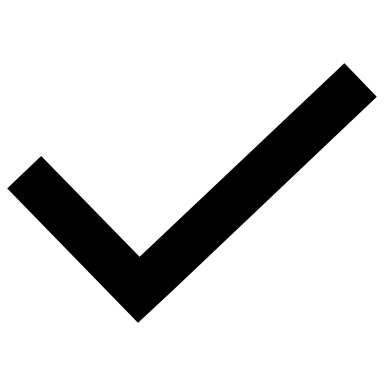 |  | 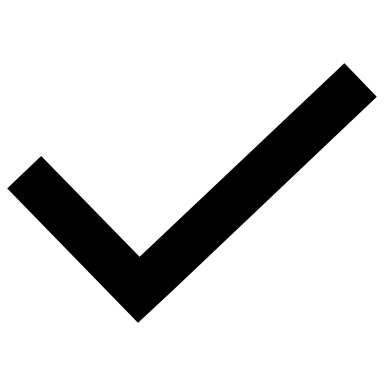 |  |  |
| Stress test with saliva samples |  |  |  |  |  | 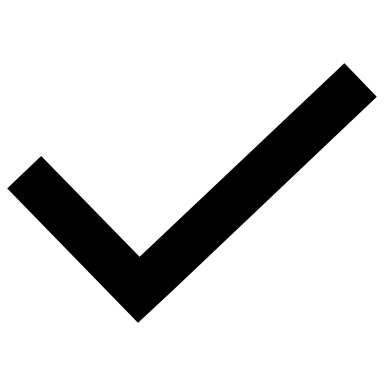 |  |
| MRI examination |  |  |  |  | 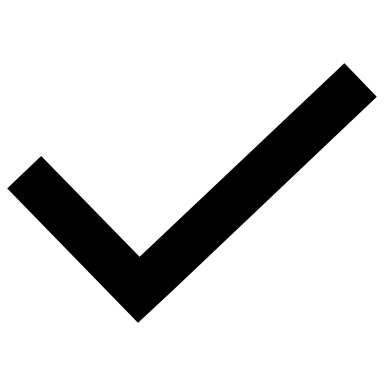 |  |  |
| Blood sample collection (including for future research) | 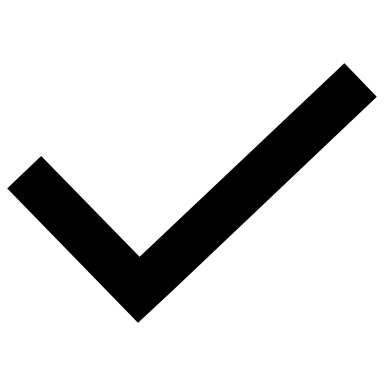 | 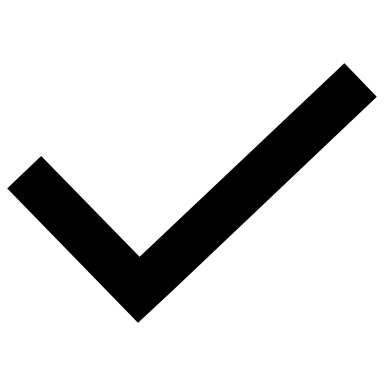 | 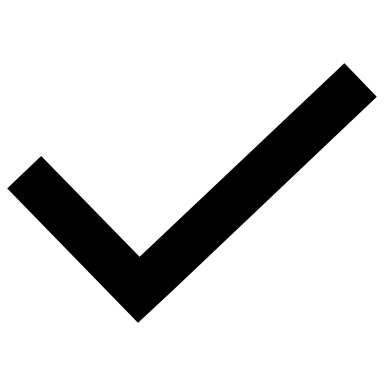 | 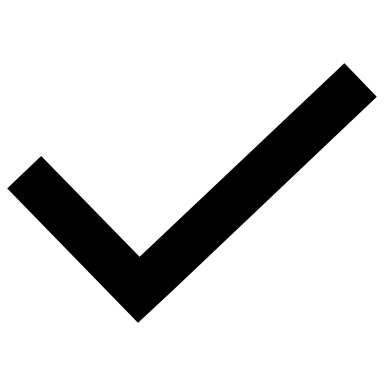 | 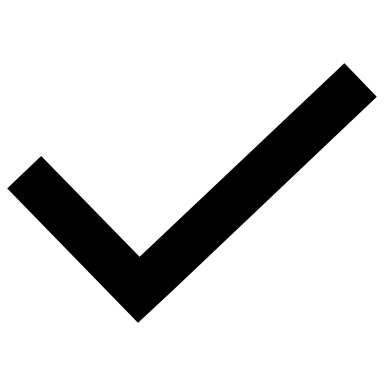 | 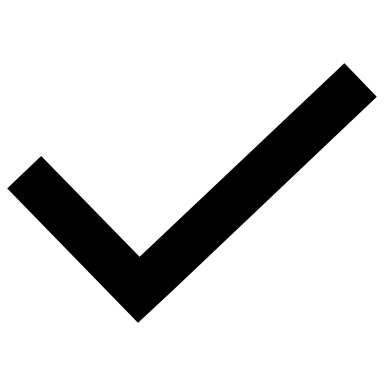 | 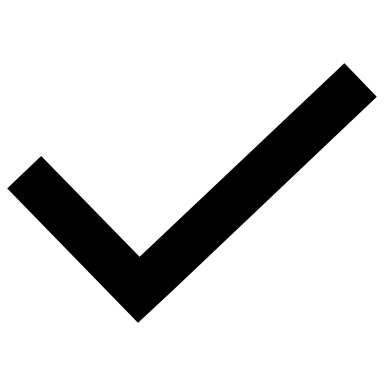 |
| Urine analysis |  | 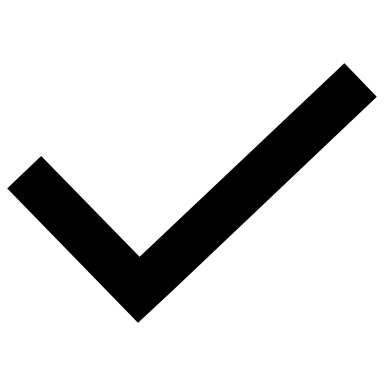 |  |  | 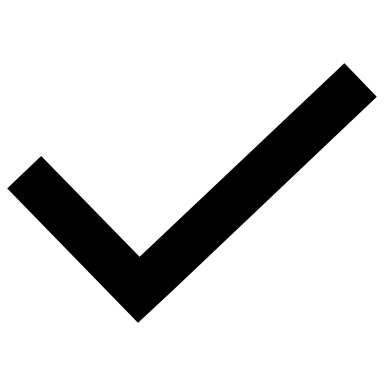 | 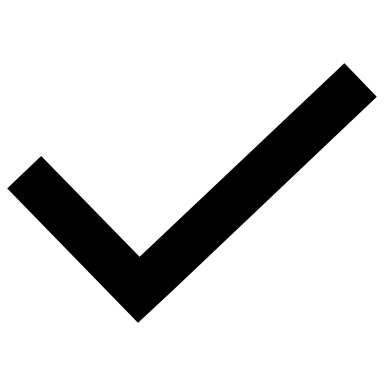 | 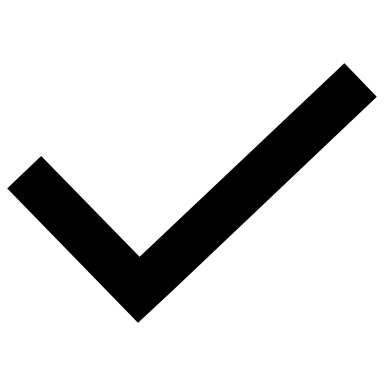 |
| Pregnancy test in urine | 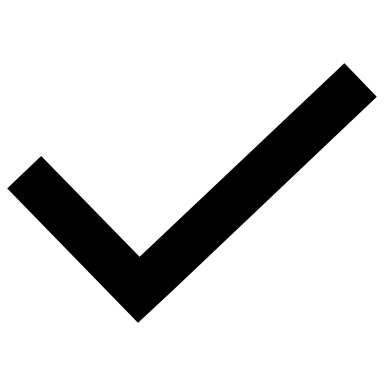 |  |  |  |  |  |  |

We will coordinate the scheduling of appointments with you and provide a comprehensive overview of the dates. Please note that the appointments are not easily rescheduled. If, however, you find it necessary to reschedule an appointment due to important reasons, we kindly request you to inform us promptly.

## When does participation in the study end?

For you, participation in the study extends over a period of **28 days**, concluding with the study visit on the 28th day. You have the option to terminate your participation at any time (-> Chapter 5.4). Providing an explanation for discontinuing your participation is not mandatory. If you choose to conclude your participation early, please discuss it with your investigator.

Even if you decide to end your participation prematurely, we will continue to provide medical treatment and care to the best of our ability in accordance with current standards (-> Chapter 5.4 for alternative treatment options). In such cases, a final examination will be conducted for your safety, and any remaining study medication should be returned to us.

Should you opt to discontinue the study early, please keep your investigator informed of any changes in your health status, such as feeling worse or experiencing new symptoms. If your participation ends prematurely, we will analyze the data and samples collected up to that point, including blood values.

It's important to note that there may be instances where we might need to request you to end your participation prematurely. For example, this may be necessary if there is an urgent need for a change in your medication or if your pregnancy test results turn out positive during the study.

## What happens if you do not want to participate?

Even if you decide not to participate in this study, we are committed to providing you with medical treatment and care to the best of our ability, adhering to current standards. In the event of your decision to forego study participation, your investigator will engage in a discussion with you about alternative treatment options.

## Pregnancy

Pregnancy and breastfeeding can result in an elevated natural production of oxytocin, impacting the clinical outcomes of this study. Furthermore, oxytocin has the potential to stimulate contractions of the uterine muscles, which may lead to the initiation of labor.

For these reasons, individuals who have given birth within the past 8 weeks, are currently breastfeeding, or are pregnant or breastfeeding at the study's commencement are not eligible to participate. It is essential to address these considerations with your investigator during discussions regarding your eligibility for the study.

### For women of childbearing potential

During your participation in the study, it is important that you refrain from becoming pregnant. It is crucial to communicate your involvement in this study to your partner. Prior to commencing the study, a urine pregnancy test will be administered, and this test will be conducted regularly throughout the study. If you are currently breastfeeding, participation is not allowed.

As a precautionary measure, effective contraception must be utilized during the study, which includes:

1. A compound that suppresses ovulation, available in the form of a pill (commonly referred to as the "birth control pill" or "mini-pill"), injection, subcutaneous implant, patch, or vaginal ring
2. A copper or hormonal intrauterine device (IUD)
3. Contraception through the use of a condom

In the event of pregnancy during the study, it is imperative that you promptly notify your investigator. Subsequently, the investigator will engage in discussions with you and your partner to determine the necessary steps forward.

# Risks, burdens and side effects

## What risks and burdens can occur?

Participating in this study involves potential risks and burdens, which is inherent in any medical treatment. Some risks are well-documented, while others may still be unknown, as is typical in research studies. **Chapter 6.2** provides a list of the most common and severe risks, and it's important to note that many side effects are medically treatable. Throughout the study, we will keep you informed about any new findings related to risks and side effects.

The study medication, oxytocin, is a well-established drug in gynecology and obstetrics, frequently utilized in these fields. Oxytocin as a nasal spray has been employed in numerous studies, and the planned dosage for this study has been deemed safe based on previous research.

In addition to the medication, there are inherent risks associated with the medical examinations conducted in this study, some of which may be familiar to you. A list of examination-related risks is available in **Chapter 6.3** for your reference.

## The most common and severe risks associated with the study medication

You will find information here about the most common and severe side effects that we already know of. For this purpose, we use the following descriptions:

| Very frequent | We find the side effect in more than 10 out of 100 people (more than 10%). |
| --- | --- |
| Frequent | We find the side effect in 1 to 10 out of 100 people (1%-10%). |
| Occasionally | We find the side effect in 1 to 10 out of 1‘000 people (0.1%-1%). |
| Rarely | We find the side effect in 1 to 10 out of 10‘000 people (0.01%-0.1%). |
| Very rarely | We find the side effect in less than 1 person out of 10‘000 (less than 0.01%). |

Frequent side effects include:

- Nasal irritation
- Headaches
- Inner restlessness
- Pulse irregularities

Further side effects include:

- Uterine cramps (occasionally)
- Skin rash (rarely)
- Allergic reactions involving difficulty breathing, drop in blood pressure, circulatory collapse (rarely)
- Increased blood pressure (very rarely)

Because Desmopressin and oxytocin share structural similarities and both medications address the symptoms of excessive urine output, it is conceivable that you may require a reduced dosage of Desmopressin. It's crucial to be aware that the concurrent use of Desmopressin could potentially result in hyponatremia (low blood sodium levels). However, you will be provided with instructions for the proper intake of these medications. Furthermore, your sodium levels (blood salt levels) will be monitored on three separate occasions: at the beginning, middle, and end of the study.

## Risks and burdens from examinations in the study

We conduct various medical examinations for this study (-> Chapter 5.2). While these examinations are established procedures, they may pose certain risks and burdens, potentially causing discomfort or leading to unwanted side effects. In the context of this study, the following risks and burdens are identified:

- Blood sampling: Puncture sites resulting from venipuncture using the venous catheter may cause minor hematomas that typically heal within a few days. In rare cases, there may be occurrences of vein closure or inflammation at the puncture site. If you experience any of these issues, please inform us immediately.
- Questionnaires: Participants are not obligated to answer any questions if they choose not to. If the questioning causes emotional strain or if there is a need for information or support, psychologically competent professionals are consistently available to provide informational support, assistance, and relieving conversations.
- Functional MRI: No harmful side effects of magnetic resonance imaging (MRI) are known. Contrast agents are not necessary for this type of examination.

# Financing and compensation

The study is financially supported through an endocrinology fund by Professor Dr. M. Christ-Crain. The researchers involved in the study do not receive any direct financial benefits from its execution. Your participation in the study will not incur any additional costs for you or your health insurance. Any travel and accommodation expenses resulting from participation will be reimbursed.

The outcomes of this study may contribute to the development of a new medication for a specific patient group. However, it's essential to note that your participation in the study does not entail any ownership stake or direct financial benefit in this regard.

# Results from our study

The investigator will keep you informed throughout the study about any new findings that may impact the study's benefits or your safety, and your consent to participate. This information will be conveyed both verbally and in writing.

In the case of incidental findings, such as those arising from laboratory tests, which could aid in preventing, detecting, or treating existing or anticipated future illnesses, you will be notified. If you prefer not to be informed (exercising your right not to know), please discuss this preference with your investigator.

At the conclusion of the study, the investigator will be happy to provide you with a summary of the overall study results if you express interest in receiving this information.

Part 3: Data protection and insurance coverage

# Protection of data and samples

We ensure the protection of your data, including information such as blood pressure and pulse from your medical history, as well as your samples, such as blood samples. Switzerland has strict legal regulations in place to safeguard your data and samples, ensuring the utmost privacy and security.

## Encryption of data and samples

In every study, data is generated through various examinations, such as blood tests and MRI findings. This data is meticulously documented, typically in electronic formats within comprehensive tables known as "data collection sheets." Importantly, all data is recorded in an encrypted manner. This encryption involves keeping personal information separate from the examination results. To achieve this, a list is maintained, assigning each person a unique code. Personal details like your name, date of birth, or address are not directly included in the data collection sheet. The key list, which links codes to individuals, is securely held at the University Hospital Basel.

In every study, data is generated through various examinations, such as blood tests and MRI findings. This data is meticulously documented, typically in electronic formats within comprehensive tables known as "data collection sheets." Importantly, all data is recorded in an encrypted manner. This encryption involves keeping personal information separate from the examination results. To achieve this, a list is maintained, assigning each person a unique code. Personal details like your name, date of birth, or address are not directly included in the data collection sheet. The key list, which links codes to individuals, is securely held at the University Hospital Basel.

At the conclusion of the study, your data will undergo complete anonymization, typically by the end of the legally prescribed storage period. This implies that identifying you without disproportionate effort will no longer be possible. Various measures, including the destruction of the code and the associated list, are employed for this anonymization process.

## Secure handling of data and samples during the study

The sponsor assumes responsibility for the secure management of your data and samples derived from this study. They are obligated to ensure compliance with relevant laws, including data protection regulations. All individuals with access to your data within the scope of the study are bound by medical confidentiality. Adherence to data protection regulations is strictly maintained, and as a participant, you have the right to access your data at any point.

Frequently, it is crucial for your primary care physician to share data from your medical history with the investigator. This requirement extends to other healthcare professionals involved in your treatment. By providing your consent at the conclusion of this document, you authorize the exchange of this information.

## Secure handling of data and samples after the study

The sponsor retains responsibility for the secure handling of your data and samples even after the study concludes. Legal requirements mandate the retention of all study documents, including data collection sheets, for a minimum of 10 years.

In cases where data and samples are stored on-site, they will be housed in a biobank (*Biobank Department Endocrinology*) for research purposes. Your data and samples may be utilized for additional investigations in the future or transferred to and employed in another database/biobank in Switzerland or abroad for investigations not yet specified. Any other database/biobank involved must adhere to the same standards as the one designated for this study. For this continued use, we kindly request you to sign another consent form at the conclusion of this document.

Following the completion of a study, the results are typically published in scientific journals. To facilitate this, the results, in encrypted form, will be forwarded to expert reviewers for assessment.

## Further use of data and samples in other future studies

Your data and samples from this study hold significant value for future research endeavors. Portions of the data and samples that haven't been fully utilized in this study may potentially be employed for other research studies. For the continued use of your genetic data and samples, we seek your additional consent. Participation in this aspect is voluntary. Please thoroughly review the supplementary consent form provided at the end of this document. If you wish to endorse further research with your data and samples in the future, kindly sign the consent form. It's important to note that even if you choose not to provide this additional consent, you can still participate in the current study.

## Rights of inspection during audits

The conduct of this study is subject to potential investigation by authorities, including the relevant ethics committee, regulatory authority *Swissmedic*, or foreign regulatory authorities. The sponsor also conducts inspections to ensure the quality of the study and its results.

As part of these inspections, a select group of specifically trained individuals may be granted access to your personal data and medical history. During these inspections, the data is not encrypted. Individuals who have access to your unencrypted data are bound by medical confidentiality

# Insurance Coverage

You are covered by insurance in the event of any harm caused by the study, specifically, harm resulting from the study medication oxytocin. This coverage is regulated by law. If you experience any health issues or damages during or after the clinical trial, please contact the responsible principal investigator (Prof. Dr. M. Christ-Crain) or reach out directly to the specified insurance company. They will take the necessary steps on your behalf.

Insurance Company: Helvetia Schweizerische Versicherungsgesellschaft AG, Dufourstrasse 40, 9001 St. Gallen.

Part 4: Conent form

I acknowledge that this consent comprises **two distinct consent forms**::

- Consent for participation in this study
- Consent for the further use of data and samples from this study in encrypted form

I have thoroughly reviewed this form and understand its contents. If there is any aspect that is unclear or if further information is needed, I am encouraged to seek clarification from the study organizers. I am aware that my written consent is essential for participation.

**Informed Consent Form for Participation in the OxyTUTION Study:**

| **BASEC-Number** | 2023 - 01010 |
| --- | --- |
| **Study title** | The OxyTUTION Study |
| **Layperson-friendly title** | Medication study with oxytocin nasal spray as a treatment option for patients with AVP deficiency [diabetes insipidus] |
| **Responsible institution** (Sponsor with address) | Prof. Dr. med. Mirjam Christ-Crain  University Hospital Basel, Petersgraben 4, 4031 Basel |
| **Location of execution** | University Hospital Basel, Petersgraben 4, 4031 Basel |
| **Investigator at the study site** | Prof. Dr. med. Mirjam Christ-Crain |
| **Participant**  **Last name and first name** in block letters  **Date of birth** |  |

- I have received verbal and written information about the study from the investigator who is signing.
- The investigator has explained to me the purpose, the procedure and the risks of the medicinal product oxytocin.
- I am voluntarily participating in the study.
- The investigator has explained to me the possible standard treatments available outside of the study.
- I have had enough time to make this decision. I will keep the written information and receive a copy of my signed consent form.
- I can end my participation at any time. I do not have to explain why. Even if I end my participation, I will continue to receive my medical care. The data and samples collected up to that point will remain stored and will be analyzed as part of the study.
- If it is considered better for my health, the investigator has the authority to remove me from the study at any time.
- I understand that my data and samples will only be shared in encrypted form. The sponsor ensures that data protection is maintained according to Swiss standards.
- If there are results and/or incidental findings directly concerning my health, I will be informed. If I do not wish to be informed, I will discuss this with my investigator.
- My primary care physician must be informed that I am participating in the study. My primary care physician can share relevant data from my medical history with the investigator. This also applies to other doctors who treat me.
- The responsible individuals from the sponsor, the ethic committee and the regulatory authority *Swissmedic* may access my unencrypted data for inspection. All these individuals are bound by medical confidentiality.

I acknowledge that Prof. Dr. med. M. Christ-Crain has procured insurance coverage. This insurance is designed to provide compensation for any harm I may experience, but only if such harm is directly attributable to the study.

| Location, Date | Last name and first name of the participant in block letters  Signature of the participant |
| --- | --- |

**Confirmation by the investigator:** I hereby confirm that I have explained to this participant the nature, significance and implications of the study. I assure that I will fulfill all obligations related to this study according to Swiss law. If I become aware of any aspects during the course of the study that could affect the participant’s willingness to participate, I will promptly inform them.

| Loaction, Date | Last name and first name of the investigator in block letters  Signature of the investigator |
| --- | --- |

**Informed Consent Form for the further use of data and samples in encrypted form**

This consent specifically does not pertain to your immediate participation in a study. The term "further use" signifies that your data and samples may be retained beyond the duration of your study involvement and employed in encrypted form for subsequent research. This could involve scenarios where, for instance, your blood sample and corresponding laboratory values are statistically analyzed in conjunction with a larger dataset or subjected to new investigations.

| **BASEC-Number** | 2023 - 01010 |
| --- | --- |
| **Study title** | The OxyTUTION Study |
| **Layperson-friendly titel** | Medication study with oxytocin nasal spray as a treatment option for patients with AVP deficiency [diabetes insipidus] |
| **Participant**  Last name and first name in block letters  Date of birth |  |

- I hereby consent to the further use of my encrypted data and samples from this study for medical research. These samples will be securely stored in a biobank and will be available for future research projects indefinitely.
- I am aware that the samples are encrypted, and the decryption key is securely stored.
- I understand that the data may be analyzed both domestically and abroad, and it may be stored in a domestic or foreign database. The samples may undergo investigation both here and abroad and could be stored in a biobank. Research institutions abroad must adhere to the same data protection standards as those applied in Switzerland.
- I understand that the data may be analyzed both domestically and abroad, and it may be stored in a domestic or foreign database. The samples may undergo investigation both here and abroad and could be stored in a biobank. Research institutions abroad must adhere to the same data protection standards as those applied in Switzerland.
- I am making this decision voluntarily and retain the right to revoke it at any time. In the event of my withdrawal, all my data will be anonymized, and my samples will be destroyed. I only need to inform my investigator and am not required to provide a reason for my decision.
- Typically, all data and samples are analyzed collectively. In the event of any highly significant incidental findings that may impact my health, I understand that I will be contacted. If I prefer not to be contacted, I will communicate this preference to my investigator.
- If applicable for anonymization: I grant permission for my data and samples to be anonymized. I comprehend that, in this scenario, I will not be informed about my individual results, and withdrawal from the research project will not be possible.

| Location, Date | Last name and first name of the participant in block letters  Signature of the participant |
| --- | --- |

**Confirmation by the investigator:** I confirm that I have explained to the participant the nature, significance and implications of the further use of samples and/or genetic data.

| Location, Date | Last name and first name of the investigator in block letters  Signature of the investigator |
| --- | --- |
