# Supplementary material for: Oxytocin substitution therapy in patients with AVP deficiency (central diabetes insipidus): study protocol of a double-blind, randomised placebo-controlled trial
Source: BMJ Open. 2026 May 4;16(5):e109940. doi: 10.1136/bmjopen-2025-109940 (PMC13141166; doi:10.1136/bmjopen-2025-109940)
Supplement: Supplementary file 3 [file bmjopen-2025-109940.reviewer_comments.pdf]

PEER REVIEW HISTORY

BMJ Open publishes all reviews undertaken for accepted manuscripts. Reviewers are asked to complete a checklist review form and are provided with free text boxes to elaborate on their assessment. These free text comments are reproduced below.

ARTICLE DETAILS

Title (Provisional)

Oxytocin Substitution Therapy in Patients with AVP Deficiency (Central Diabetes Insipidus): Study Protocol of a Double-Blind, Randomised Placebo-Controlled Trial

Authors

Atila, Cihan; Leibnitz, Svenja; Nikaj, Andi; Liechti, Matthias E; De Quervain, Dominique; Christ-Crain, Mirjam; Study Group, OxyTUTION

VERSION 1 - REVIEW

**Reviewer**	**1**
**Name**	**Boie, Sidsel**
**Affiliation**	**Randers Regional Hospital, Department of Obstetrics and Gynaecology**
**Date**	**22-Oct-2025**
**COI**	**None**

As an obstetrician, my clinical experience with AVPD is limited; my focus has therefore been on the methodological aspects of the protocol.

You have used the CONSORT checklist, which is intended for reporting RCTs. I suggest using the SPIRIT checklist when writing the protocol.

Consider mentioning patient and public involvement.

Author list:

Consider mentioning the names of individuals included in the OxyTUTION study group, e.g. in the acknowledgements section.

Background:

Consider providing information on the rationale for intranasal administration of oxytocin, and include some thoughts on the appropriate oxytocin dosage.

Screening and recruitment:

Please elaborate on who will be responsible for informing eligible participants, and what resources are required to inform them about the study.

Randomisation:

Please elaborate on whether different block sizes are used for randomisation, and if so, specify the block sizes.

Please describe whether any stratification is applied during randomisation.

Indicate when the randomisation takes place, to allow readers to understand the potential for post-randomisation dropouts. Consider linking this to the study flow in Figure 2.

Data entry and quality control:

Principles of Good Clinical Practice (GCP) and GCP monitoring are not mentioned — please consider including this.

Statistical analysis:

How will missing data be handled?

Blinding and dispensing:

Please elaborate on which study personnel are blinded, and how blinding is achieved. Consider rephrasing this section, as it is currently unclear.

No information is provided on medication storage. Should the nasal spray be kept refrigerated, or is ambient storage sufficient?

Intervention/control:

There is no information on criteria for discontinuation, unblinding, or modification of the allocated treatment.

No details are provided on how adherence to the allocated treatment will be monitored or supported.

Interim analysis:

Consider including information about the composition of the DSMB.

Serious adverse events should be described in more detail.

No stopping rules are mentioned. Interim analyses require pre-specified statistical methods to avoid compromising the integrity of the study.

Clarify who will have access to the interim analysis results.

Ethics approval:

The trial must be registered prior to recruitment.

According to the record history on ClinicalTrials.gov, no protocol changes were made after recruitment started.

Please include all relevant information regarding trial registration and ethical approvals.

It appears that the trial has been registered in CTIS with the EUCT number 2024-516813-19-00 and protocol code OxyTUTION.

**Reviewer**	**2**
**Name**	**Hagiwara, Daisuke**
**Affiliation**	**Nagoya University**
**Date**	**03-Dec-2025**
**COI**	**None**

This is a well-designed and timely study protocol addressing an important and understudied aspect of AVP-D: the psychological burden potentially related to co-existing OT deficiency. I am broadly supportive of the study. If intranasal OT improves psychological or emotional outcomes in patients with AVP-D, this trial could establish an important proof-of-concept for OT as a novel hormone replacement therapy in hypopituitarism. Overall, the protocol is clearly written, the trial design is appropriate, and the planned analyses are reasonable. I have several minor comments that mainly aim to clarify and further strengthen the rationale, outcome definitions, and safety considerations.

1. Inclusion criteria versus primary outcomes

The inclusion criterion allows participants with elevated alexithymia (TAS-20 ≥52) even when baseline anxiety is not markedly elevated, whereas, the primary endpoint relies mainly on STAI-T. Could the authors clarify the rationale for this inclusion strategy, and discuss whether this discrepancy may affect the ability to detect meaningful changes in STAI-T among participants with only modest baseline anxiety?

2. Definition and justification of the composite primary endpoint

The primary endpoint is defined as a binary “success” based on either a ≥5-point decrease in STAI-T or a ≥4-point increase in EmBody/EmFace performance. While both components are individually reasonable, combining trait anxiety and emotion recognition into a single composite endpoint may complicate interpretation, particularly if the treatment has different effects on these domains. It would be helpful to pre-specify how the authors will interpret the results if OT improves only one component (e.g. emotion recognition) but not the other (anxiety), and whether analyses of the two components as separate secondary/tertiary outcomes are planned.

3. Safety monitoring and management of sodium imbalance

The Discussion acknowledges the potential natriuretic and antidiuretic effects of OT through AVP2-receptor crosstalk and the increased prevalence of hyponatremia in desmopressin-treated AVP-D patients. The protocol mentions a safety visit around day 14 with sodium monitoring and patient education regarding hyponatremia. Given the potential for

synergistic effects of OT and desmopressin: 1) Please provide more detail on the planned schedule of serum sodium measurements (both on-site and via local providers) across the 28-day period. 2) It would be helpful to specify predefined sodium thresholds and corresponding actions (e.g. dose reduction, temporary interruption, or termination of OT and/or desmopressin), as well as how these events will be captured and reported. 3) Clarifying whether any adjustment of the background desmopressin regimen is allowed or anticipated during the trial would also be valuable, since changes in desmopressin dosing may affect both safety and the interpretation of outcomes.

4. Reference formatting

In the References section, "Reference 1" appears twice; renumbering or correction would improve clarity.

VERSION 1 - AUTHOR RESPONSE

Reviewer: 1 Dr. Sidsel Boie, Randers Regional Hospital

As an obstetrician, my clinical experience with AVPD is limited; my focus has therefore been on the methodological aspects of the protocol.
We thank Dr. Boie for her careful review and constructive comments.

You have used the CONSORT checklist, which is intended for reporting RCTs. I suggest using the SPIRIT checklist when writing the protocol.
Thank you for this important comment. We have replaced the CONSORT checklist with the SPIRIT checklist.

Consider mentioning patient and public involvement.
We agree and have now included a Patient and Public Involvement section in the main text (see pages 15-16).

Author list:
Consider mentioning the names of individuals included in the OxyTUTION study group, e.g. in the acknowledgements section.
Thank you for this suggestion. We agree and have included the names of the individuals involved in the OxyTUTION Study Group in the Acknowledgements section.

Background:
Consider providing information on the rationale for intranasal administration of oxytocin, and include some thoughts on the appropriate oxytocin dosage.
Thank you for this comment. We have expanded the Background sections to provide a rationale for the intranasal route of oxytocin administration. In addition, we now justify the

selected dosing regimen by referencing established human studies, including evidence for a non-linear dose–response relationship and the frequent use of 24 IU per administration, which informed our choice of a total daily dose of 48 IU administered in two divided doses (see pages 4-5).

Screening and recruitment:

Please elaborate on who will be responsible for informing eligible participants, and what resources are required to inform them about the study.

We have updated the Screening, Recruitment and Informed Consent section (see pages 5-6).

Randomisation:

Please elaborate on whether different block sizes are used for randomisation, and if so, specify the block sizes. Please describe whether any stratification is applied during randomisation.

We have clarified the randomisation procedure in the manuscript. Randomisation will be performed using block randomisation with a fixed block size of four, and no stratification factors are applied (see pages 6-7).

Indicate when the randomisation takes place, to allow readers to understand the potential for post-randomisation dropouts. Consider linking this to the study flow in Figure 2.

Randomisation takes place immediately after completion of the screening visit, once a participant has been confirmed to fulfil all eligibility criteria and has provided written informed consent. We described this on pages 6-7 and linked to Figure 2.

Data entry and quality control:

Principles of Good Clinical Practice (GCP) and GCP monitoring are not mentioned — please consider including this.

We have now included statements in the main text confirming that the study will be conducted in accordance with the principles of Good Clinical Practice (ICH-GCP) and that GCP-compliant monitoring will be performed throughout the trial (see pages 5 and 16).

Statistical analysis:

How will missing data be handled?

We have added a description to the main text, including the use of best- and worst-case imputation for missing primary outcome data (see page 15).

Blinding and dispensing:

Please elaborate on which study personnel are blinded, and how blinding is achieved. Consider rephrasing this section, as it is currently unclear.

We have revised and clarified the Blinding and Dispensing section to specify which study personnel are blinded (participants, study physicians, study nurses, study psychologists, and all outcome assessors) and to describe how blinding is maintained, including the use of unblinded personnel solely for medication storage and dispensing and procedures to prevent unblinding due to differences in container systems (see page 7).

No information is provided on medication storage. Should the nasal spray be kept refrigerated, or is ambient storage sufficient?

We have added details on medication storage, specifying that the investigational product is stored refrigerated at 2–8 °C before opening and may be kept at room temperature (15–25 °C) after opening, in accordance with manufacturer recommendations (see page 8).

Intervention/control:

There is no information on criteria for discontinuation, unblinding, or modification of the allocated treatment. No details are provided on how adherence to the allocated treatment will be monitored or supported.

Thank you for this comment. We have expanded the Intervention/Control section to specify criteria for treatment discontinuation or modification as well as the procedures for emergency (see pages 7-8).

Interim analysis:

Consider including information about the composition of the DSMB. Serious adverse events should be described in more detail.

Thank you for this suggestion. We have added details to the manuscript specifying the composition of the DSMB, which includes two independent neuroendocrinologists, one patient representative, and one data manager or statistician (see page14). We have expanded the description of serious adverse events, specifying that hyponatremia is the main anticipated serious adverse event, and we now detail its grading, monitoring schedule, and predefined management procedures in the manuscript.

No stopping rules are mentioned. Interim analyses require pre-specified statistical methods to avoid compromising the integrity of the study. Clarify who will have access to the interim analysis results.

We have clarified that a pre-specified safety signal is defined: if five or more additional cases of severe hyponatremia occur in the oxytocin group compared with the placebo group, the DSMB will issue a recommendation for trial modification, which may include protocol amendments, enhanced safety monitoring, temporary interruption, depending on the clinical context. Access to interim safety data is restricted to the independent

DSMB, while investigators and the study team remain blinded, ensuring that trial integrity and blinding are not compromised.

Ethics approval:
The trial must be registered prior to recruitment.
According to the record history on ClinicalTrials.gov, no protocol changes were made after recruitment started.
The trial was registered prior to the start of participant recruitment to ClinicalTrials.gov. Only minor administrative updates required by new regulatory regulations were made thereafter, and one exclusion criterion was recently removed. These changes will be updated in the trial registry as soon as possible.

Please include all relevant information regarding trial registration and ethical approvals. It appears that the trial has been registered in CTIS with the EUCT number 2024-516813-19-00 and protocol code OxyTUTION.
We have updated the manuscript to include all relevant trial registration and ethical approval details, specifying registration in ClinicalTrials.gov (NCT06036004) and in the Clinical Trials Information System (CTIS) with EUCT number 2024-516813-19-00 (protocol code: OxyTUTION), as well as approval by the competent ethics committees and regulatory authorities in Switzerland and the European Union (see page 5).

Reviewer: 2 Dr. Daisuke Hagiwara, Nagoya University

This is a well-designed and timely study protocol addressing an important and understudied aspect of AVP-D: the psychological burden potentially related to co-existing OT deficiency. I am broadly supportive of the study. If intranasal OT improves psychological or emotional outcomes in patients with AVP-D, this trial could establish an important proof-of-concept for OT as a novel hormone replacement therapy in hypopituitarism. Overall, the protocol is clearly written, the trial design is appropriate, and the planned analyses are reasonable. I have several minor comments that mainly aim to clarify and further strengthen the rationale, outcome definitions, and safety considerations.
We sincerely thank Dr. Hagiwara for his supportive and constructive review.

1. Inclusion criteria versus primary outcomes
The inclusion criterion allows participants with elevated alexithymia (TAS-20 ≥52) even when baseline anxiety is not markedly elevated, whereas, the primary endpoint relies mainly on STAI-T. Could the authors clarify the rationale for this inclusion strategy, and discuss whether this discrepancy may affect the ability to detect meaningful changes in STAI-T among participants with only modest baseline anxiety?

Thank you for this thoughtful comment. We agree that allowing inclusion of participants with elevated alexithymia in the absence of markedly increased baseline anxiety may limit the ability to detect large changes in STAI-T within this subgroup. However, this inclusion strategy reflects the dual conceptual focus of the study on anxiety and socio-emotional processing, rather than anxiety alone. Importantly, the primary endpoint does not rely solely on STAI-T but is defined as a composite outcome that also includes performance on the EmBody/EmFace task. While STAI-T is a well-established and validated measure of trait anxiety, emotion perception and processing are assessed using a computerized task that is more sensitive to subtle changes in emotional perception and valence than questionnaire-based instruments. The choice of this dual primary endpoint was guided by the physiological and clinical evidence available for oxytocin deficiency: prior data strongly support effects of oxytocin on emotion recognition, while increased anxiety is also a consistent and clinically relevant feature. We therefore consider the clinical phenotype of oxytocin deficiency to represent a mixed picture of heightened anxiety and impaired emotion recognition, which is appropriately captured by the chosen inclusion criteria and endpoint strategy.

2. Definition and justification of the composite primary endpoint

The primary endpoint is defined as a binary "success" based on either a ≥5-point decrease in STAI-T or a ≥4-point increase in EmBody/EmFace performance. While both components are individually reasonable, combining trait anxiety and emotion recognition into a single composite endpoint may complicate interpretation, particularly if the treatment has different effects on these domains. It would be helpful to pre-specify how the authors will interpret the results if OT improves only one component (e.g. emotion recognition) but not the other (anxiety), and whether analyses of the two components as separate secondary/tertiary outcomes are planned.

We thank the reviewer for this important comment and agree that combining trait anxiety and emotion recognition into a composite endpoint may complicate interpretation if oxytocin exerts differential effects on these domains. The rationale for the composite primary endpoint reflects the hypothesis that oxytocin may improve psychological functioning in AVP-D through complementary mechanisms, both affective state (anxiety) and emotional processing. We agree that oxytocin may preferentially affect one domain over the other. Therefore, we will pre-specify that improvement in either component will be interpreted as evidence of a domain-specific treatment effect rather than as a uniform effect across all psychological outcomes. Importantly, the two components of the composite endpoint (STAI-T and EmBody/EmFace performance) will also be analyzed separately as secondary outcomes, allowing domain-specific interpretation of treatment effects.

3. Safety monitoring and management of sodium imbalance

The Discussion acknowledges the potential natriuretic and antidiuretic effects of OT through AVP2-receptor crosstalk and the increased prevalence of hyponatremia in desmopressin-treated AVP-D patients. The protocol mentions a safety visit around day 14 with sodium monitoring and patient education regarding hyponatremia. Given the potential for synergistic effects of OT and desmopressin: 1) Please provide more detail on the planned schedule of serum sodium measurements (both on-site and via local providers) across the 28-day period. 2) It would be helpful to specify predefined sodium thresholds and corresponding actions (e.g. dose reduction, temporary interruption, or termination of OT and/or desmopressin), as well as how these events will be captured and reported. 3) Clarifying whether any adjustment of the background desmopressin regimen is allowed or anticipated during the trial would also be valuable, since changes in desmopressin dosing may affect both safety and the interpretation of outcomes.

Thank you for these important safety-focused comments. We agree that potential synergistic antidiuretic effects of OT and desmopressin warrant a clearly predefined monitoring and action plan, and we will expand the protocol accordingly (see page 13):

- *Schedule of serum sodium monitoring: We will specify a structured monitoring schedule with serum sodium at baseline (pre-treatment), Day 14 (±2 days; on-site safety visit), and Day 28 (±2 days; end-of-treatment).*
- *Predefined sodium thresholds and actions for desmopressin use: We will include an explicit algorithm, for example:*
    - *Na 130–134 mmol/L: The patient will again be instructed in detail by our investigator on how to perform the 'Desmopressin Escape' method. We will follow up on plasma sodium within the next 7 days.*
    - *Na 125–129 mmol/L: Procedure see above. However, plasma sodium control is done earlier, after 2-3 days.*
    - *Na <125 mmol/L: The patients are monitored in the hospital until normalization of the sodium value/symptomatic improvement after detailed assessment by the investigator.*

We will also specify that hyponatremia events (including severity, symptoms, interventions, and outcomes) will be recorded as AEs/SAEs, including whether they led to dose interruption/discontinuation and any desmopressin changes, and will be reported in the safety analysis.

- *Desmopressin regimen during the trial: Hyponatremia is a common complication of desmopressin therapy in AVP-D, affecting up to 27% of outpatients. To mitigate this risk, all participants will be instructed to use the "desmopressin escape method," allowing breakthrough aquaresis before the next dose, which has been shown to significantly reduce hyponatremia compared with rigid dosing. The OXT dose will remain stable throughout the study, while desmopressin dosing may be adjusted if needed; patients will be educated on symptoms of hyponatremia, and daily desmopressin dose and fluid intake will be documented in a diary.*

4. Reference formatting
In the References section, "Reference 1" appears twice; renumbering or correction would improve clarity.
Thank you for noting this. We corrected the duplicate listing.

VERSION 2 - REVIEW

**Reviewer**	**1**
**Name**	**Boie, Sidsel**
**Affiliation**	**Randers Regional Hospital, Department of Obstetrics and Gynaecology**
**Date**	**04-Feb-2026**
**COI**	

Thank you for the opportunity to review your resubmission of the study protocol. The protocol has significantly improved since the initial submission.

To further assist the reader, you may consider including a few illustrations, for example:

1. The participant timeline included in the participant information

2. A CONSORT flowchart illustrating the expected inclusion and randomisation

---

**Reviewer**	**2**
**Name**	**Hagiwara, Daisuke**
**Affiliation**	**Nagoya University**
**Date**	**03-Feb-2026**
**COI**	

The authors have responded appropriately to my concern.

VERSION 2 - AUTHOR RESPONSE

Reviewer 1: Dr. Sidsel Boie, Randers Regional Hospital

Comments to the Author:
Thank you for the opportunity to review your resubmission of the study protocol. The protocol has significantly improved since the initial submission.

To further assist the reader, you may consider including a few illustrations, for example:

1. The participant timeline included in the participant information

Thank you for pointing that out. The patients' timeline is already shown in Figure 2. We are not sure whether an additional figure with the same content would be benefitial, so we'd prefer to leave it as it is

2. A CONSORT flowchart illustrating the expected inclusion and randomisation

Thank you for this valuable input. A CONSORT flowchart has been added to the appendix.

Reviewer 2 : Dr. Daisuke Hagiwara, Nagoya University

Comments to the Author:
The authors have responded appropriately to my concern.
